# Assessing the efficacy of probiotics in augmenting bovine reproductive health: an integrated *in vitro*, *in silico*, and *in vivo* study

**DOI:** 10.3389/fmicb.2023.1137611

**Published:** 2023-05-18

**Authors:** Purva Gohil, Bhavya Nanavati, Kajal Patel, Vishal Suthar, Madhvi Joshi, Deepak B. Patil, Chaitanya G. Joshi

**Affiliations:** ^1^Gujarat Biotechnology Research Centre, Gandhinagar, Gujarat, India; ^2^Directorate of Research, Kamdhenu University, Gandhinagar, Gujarat, India

**Keywords:** bovines, microbiota, probiotics, *in vitro*, *in silico*, *in vivo* performance

## Abstract

The aim of this study was to isolate and characterize bovine-vaginal probiotics genotypically and phenotypically using *in silico* and evaluate their *in vivo* performance in buffaloes with endometritis. For the *in vitro* isolation and characterization, vaginal swabs were collected from 34 cows and 17 buffaloes, and 709 primary bacterial isolates with probiotic activity were obtained using MRS agar media. Two isolates *Lactiplantibacillus plantarum* KUGBRC (LPKUGBRC) and *Pediococcus pentosaceus* GBRCKU (PPGBRCKU) demonstrated optimum *in vitro* probiotic activities as compared to *Lacticaseibacillus rhamnosus* GG including, acid production, secretion of fatty acids and exopolysaccharide, cell surface hydrophobicity, self-aggregating and co-aggregating capacity with pathogens, anti-microbial activity and bacteriocin-like compounds against pathogens *Escherichia coli* and *Staphylococcus aureus* in cell-free supernatant and absence of hemolytic activity. Their phenotypic capacity was confirmed by analyzing the whole genome sequencing data and identifying genes and pathways associated with probiotic properties. These probiotic isolates have shown no virulence genes were discovered in their genomic study. *In vivo* study of 92 buffaloes suffering from clinical endometritis with purulent cervico-vaginal mucus (CVM) were randomly allocated 40 × 10^8^ CFU/ml LPKUGBRC and PPGBRCKU and 40 ml Normal saline. The LPKUGBRC reduced the duration between administration of probiotic to induction of healthy estrus significantly. However, no effect was observed on pregnancy rate. These results suggest that LPKUGBRC and PPGBRCKU probiotic bacteria demonstrate probiotic efficiency and adaptability. Further sourced from the same niche as the targeted infection, they offer a distinct advantage in targeting the specific microbial population associated with endometritis. The findings of this study highlight the potential of LPKUGBRC and PPGBRCKU probiotics in treating endometritis and suggest further exploration of their clinical applications.

## Graphical Abstract

Representing the role of probiotics to augment fertility in bovines and combating pathogenic infections (created with BioRender.com). 
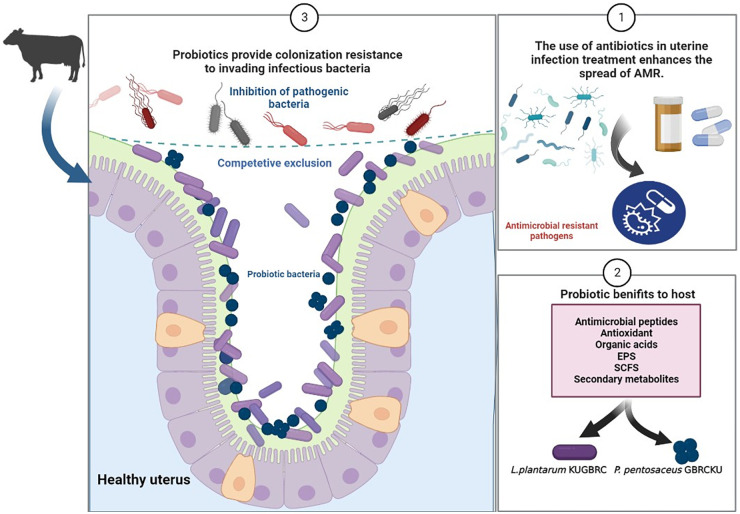

## 1. Introduction

Endometritis is considered a prototypical prevalent infection caused by inflammation of the endometrial lining in farm animals, lasting up to 21 days after calving (Sheldon et al., 2008). In 80–90% of postpartum bovines, bacterial contamination is a significant contributor to these non-specific uterine infections (Williams et al., 2008). Failure to resolve the infection can jeopardize uterine function, with pathogenic bacteria persisting for at least 2–3 weeks after postpartum developing clinical endometritis in 10–20% of dairy cattle, thereby reducing milk yield (Gilbert et al., 2005; Williams et al., 2008), resulting in a 20% lower conception rate, a 30-day longer median open period and 3% more animals culled for infertility (Gilbert et al., 2005; Sheldon et al., 2008). It has been documented that the vaginal microbiota is responsible for affecting fertility (Mahesh et al., 2021).

Postpartum bovine uterus is usually contaminated with a range of culturable and unculturable microorganisms, but this is not always consistently associated with clinical diseases. The cultivable bacteria causing infection encompass *Escherichia coli*, *Trueperella pyogenes*, *Fusobacterium necrophorum*, *Prevotella melaninogenica*, and *Porphyromonas species* (Mandhwani et al., 2017). Recent studies on non-culturable approaches suggest the presence of Bacteroides and Fusobacterium may develop reproductive diseases postpartum (Wang et al., 2018). While another study reports the low ratio of Firmicutes to Bacteroidetes causes dysbiosis of the microbial community in cows who develop endometritis and metritis (Galvão and Bicalho, 2019). Excess growth of opportunistic bacteria from the phylum Bacteroidetes and Fusobacteria in comparison with Firmicutes, contributes to the development of bovine reproductive diseases (Wang et al., 2018). The reported pathogens or opportunistic pathogens are believed to ascend from the vagina or from the environment or feces when the cervix opens or by hematogenous route in the uterus (Jeon et al., 2017). Significantly, the reproductive microbiota of bovines are influenced by sex steroid hormones, physio-pathological circumstances, environment, and nutrition (Mahesh et al., 2020; Gohil et al., 2022; Suthar et al., 2022).

Researchers estimated that the annual cost to treat a uterine disease within the European Union is €1.4 billion, and $650 million in the United States (Benzaquen et al., 2007). Apparently, the presumed annual cost to treat uterine diseases in the bovine industry in the state of Gujarat, India is around Rs. 4.7 Cr per year. Considering polymicrobial infection, many research groups and workers have tested a wide spectrum and/or combination of antibiotics and developed antibiotic standard operating procedures. However, outcomes of these antibiotics or antibiotic protocols have varying or limited results (67–77%) (Drillich et al., 2001). Furthermore, the traditional antibiotic approach leads to anti-microbial resistance (AMR), which is a global public health concern (Call et al., 2008). AMR microorganisms can withstand antimicrobial treatment strategies, leading traditional therapies to fail and infections to persist, increasing the risk of infection spreading to others. Many studies have tested hormone therapy (i.e., PGF2α, PGE, and oxytocin), iodine solution, ecbolic, ethnoveterinary medicine, herbal, and ayurvedic remedies to overcome this problem. However, the outcomes are inconsistent, subjective, and spendthrift, and they have not been generally adopted by veterinary clinicians (Hammon et al., 2006).

On the other hand, a new line of research spawned with probiotic agents as an alternative to antimicrobial compounds. Probiotics are living microorganisms that, when administered in adequate amounts, confer health benefits on the host (Fuller, 1989). Probiotics can be found in a variety of sources, including fermented foods, dietary supplements, and human and animal microbiota. The most common groups of probiotics are lactic acid bacteria (LAB) and bifidobacteria. Other groups of probiotics include streptococci, lactococci, and propionibacteria (Nami et al., 2018). A number of LAB strains, species belonging to the genera *Lactobacillus*, *Bifidobacterium*, and *Enterococcus*, are considered beneficial to the host and have, thus, been used as probiotics (Uyeno et al., 2015). They have shown the ability to improve immunological responses in the host, such as increasing the number of immune cells and modulating cytokine or lymphocyte proliferation (Klaenhammer et al., 2012; de Moreno de LeBlanc and LeBlanc, 2014; Muna and Adel, 2014).

Probiotic strains have been widely applied in the food and drug industries due to their potential health benefits. For example, encapsulation techniques have been developed to improve the survival rate and storage stability of probiotics in dairy products (Nami et al., 2017) and to enhance the resistance of fish against bacterial infections (Kahieshesfandiari et al., 2021; Nami et al., 2022). Moreover, probiotic-enriched potato chips have been developed using novel microencapsulation matrices to improve their sensory characteristics and shelf-life (Kiani et al., 2021). Furthermore, LAB isolated from colostrum have been evaluated for their probiotic potential and safety, indicating their potential application in functional foods and nutraceuticals (Haghshenas et al., 2021). In human studies, probiotics administered in the vaginal route have been shown to minimize the incidence of vaginal infections in women (Falagas et al., 2007; Cribby et al., 2008; Borges et al., 2014). Several studies support the notion of decreasing uterine infections in dairy cattle by administering LAB (Zhang et al., 2022). However, there is a dearth of scientific information on buffaloes and Zebu cattle regarding the utilization of probiotics to lower the incidence of uterine infections and improve reproductive performance (Genís et al., 2018; Suthar et al., 2022). While probiotics have been extensively studied in animal production for gastrointestinal-related diseases, research on their use for reproductive tract infections remains limited. This study characterized two lactobacillus strains isolated from the reproductive tract of healthy buffaloes, both phenotypically and genotypically, to evaluate their potential use as a treatment for endometritis. In addition to *in vitro* assays, we also conducted *in vivo* studies to evaluate the efficacy of these probiotic candidates in a live animal model. The objective of this study was to isolate and characterize two lactobacillus strains from the reproductive tract of healthy buffaloes, both phenotypically and genotypically.

The overarching aim of this study was to expand the current knowledge on the use of probiotics for reproductive tract infections, particularly in the context of treating endometritis in bovines. Specifically aims of this study were to: (1) isolate and evaluate *in vitro* performance of vaginal probiotics strains from the reproductive tract of healthy buffaloes; (2) characterize probiotics with *in silico* approach phenotypically and genotypically; and (3) sought the potential of these isolated probiotics using *in vivo* approach in buffaloes.

## 2. Materials and methods

### 2.1. *In vitro* characterization of lactobacillus bacteria

#### 2.1.1. Sample collection

For a collection of healthy uterus probiotic cultures, a total of 34 cows and 17 buffaloes were selected for this experiment from the Ambulatory Clinic of Kamdhenu University, Sanoda, Taluka Dehgam, Gandhinagar, Gujarat, India. The cows and buffaloes reported for artificial insemination (AI) with apparently healthy and with optimum body condition score (2.5–3), without any reproductive deformities or infection (white slide test negative) were enrolled for the study. The age of the selected animals ranged from 4 to 8 years (4.8 ± 3.5 years; median ± SD years) with bodyweight from 310 to 510 kg (359 ± 110 kg; median ± SD kg). As the animals were reported for AI, no approval for IAEC is needed for the experiment. Our (Gohil et al., 2022) research outlines the process of collecting vaginal swab samples from the fornix of the vagina close to the external orifice of the uterus. Indeed, samples from each cow and buffalo were collected from the fornix of vagina using the uterine swab (part no. 17214/2951/2950; Minitub GmBH, Hauptstrasse 41, 84184, Tiefenbach, Germany). Before taking the vaginal samples, external genitalia were washed with 4% chlorhexidine (Excelle, DRE Veterinary, India). The samples were transported maintaining a 4°C temperature and subsequently subjected to isolation of probiotic cultures.

#### 2.1.2. Bacterial strains and culture conditions

Microorganisms isolated from vaginal swab samples were suspended in 4 ml of 1× PBS buffer and incubated at 37°C with intermittent mixing using a vortex. Dilutions were prepared and then isolation was carried out using the spread plate and pour plate methods on microbiological media (MRS; recommended for the growth of the Lactobacilli group of bacteria). Pure bacterial cultures were obtained through sub-culturing and stored at −80°C in 25% glycerol for future experiments. Primary identification was performed based on morphological characteristics, Gram staining, and catalase reaction results. The microorganisms were identified based on the guidelines provided by Bergey’s Manual of Systematic Bacteriology (Kandler, 1986). To evaluate and compare the probiotic properties of our bacterial isolates, we chose a well-established and widely recognized probiotic strain, *Lacticaseibacillus rhamnosus* GG (LRGG).

#### 2.1.3. Hydrophobicity, auto-aggregation, and co-aggregation abilities

Bacterial cultures were grown overnight in MRS broth incubated at 37°C with shaking. Bacterial cells were harvested by centrifugation (2,000 × *g*, 15 min, 4°C), washed twice in PBS buffer (NaCl = 8 g/L; KCl = 200 mg/L; Na_2_HPO_4_ = 1.44 g/L; KH_2_PO_4_ = 245 mg/L pH 7.4 ± 0.2) and finally, OD_600_ was adjusted to 0.55–0.60 in the same buffer. Auto-aggregation was determined at initial time (0 h) and after 2 h of incubation of these isolated bacterial cultures by measuring absorbance at 600 nm. The percentage of the auto-aggregation assay is expressed as:


$$\%Autoaggregation=\left(1-\left(\frac{A\,0}{A\,t}\right)\right)*100$$

*A0*, the optical density at 0 h; *At*, the optical density after 2 h.

To assess the co-aggregation, an equal volume of each pathogen (*Staphylococcus aureus* and *E. coli*) was mixed and incubated at room temperature. Absorbance was measured at 600 nm at 0 h and after 2 h of incubation and the percentage of co-aggregation determined as:


$$\%Coaggregation=\left[\frac{A\,x+A\,y}{2}\right]-\frac{A\,\left(x+y\right)\,\left(A\,x-A\,y\right)}{2}*100$$

*Ax* and *Ay* represent absorbance of probiotic isolates and pathogenic bacteria individually in the control tube and *A*(*x* + *y*) represents the absorbance of the mixture of probiotic isolate with pathogenic bacteria.

The cell surface hydrophobicity percentage of bacterial isolates toward hydrocarbons such as chloroform, ethyl acetate, and xylene was evaluated following the protocol outlined by Chen et al. (2014). Bacterial cell pellets were harvested as described previously which was followed by centrifugation at 2,000 × *g*, 15 min and 4°C, and then resuspended in a PBS buffer. Each hydrocarbon was added to the cell suspension in the proportion of 1:3 respectively, vortexed for 5 min, and incubated for 20 min at room temperature. Further, the OD was measured using the aqueous phase at 600 nm (At). The percentage of the cell surface hydrophobicity (% H) was calculated as follows:


$$\%Hydrophobicity=\left(1-\left(\frac{A\,t}{A\,0}\right)\right)*100$$

*A0*, the optical density at 0 h; *At*, the optical density after 20 min.

#### 2.1.4. Antimicrobial activity by well diffusion assay and partial bacteriocin purification from cell-free supernatant

In order to investigate the mechanism of inhibition of pathogenic bacterial growth, an agar well diffusion assay was performed. Firstly, LAB strains were cultured in MRS broth for 48 h at 37°C and their cell-free supernatant (CFS) was obtained by centrifugation at 5,000 × *g* for 10 min at 4°C. The obtained CFS was then concentrated at 65°C for 1.5 h using a vacuum concentrator. Overnight cultures of indicator microorganisms, *E. coli* and *S. aureus*, were washed twice in PBS solution and resuspended in fresh PBS solution to obtain a concentration of approximately 10^7^ cells/ml (OD_600_ ∼ 0.25). 100 μl cell suspension was spread onto nutrient agar plates, and wells with 8 mm diameter were created in the agar. A total of 100 μl of concentrated CFS was added to each well, and fresh MRS broth was used as a negative control. The plates were then incubated aerobically at 37°C for 24 h, and the inhibitory activities were calculated by measuring the diameter of the zones of inhibition around the wells (Akabanda et al., 2014).

Quantification was conducted by doing broth dilution once activity was observed in the CFS agar well diffusion assay. To determine the minimum inhibitory concentration (MIC) of probiotic cells against pathogens, the broth dilution procedure was employed, using CFS obtained from probiotic cells. The concentrated CFS was tested against the pathogens to evaluate its effectiveness in inhibiting their growth. The pathogen inoculum was prepared by growing the pathogen in a nutrient broth media until it reached the logarithmic phase. The cells were collected by centrifugation and resuspended in saline to obtain a cell suspension with a turbidity equivalent to a 0.5 McFarland standard. The CFS was prepared by growing the probiotic cells in MRS medium for 48 h, and the cells were removed by centrifugation. The supernatant was filtered through a 0.22-μm filter to obtain the cell-free supernatant. The CFS was diluted with the growth medium to obtain a series of twofold dilutions, ranging from 1:2 to 1:32. Inoculation in the microtiter plate involved adding equal volumes of the diluted CFS and pathogen suspension to each well in the dilution series. The microtiter plate was then incubated at 37°C for 24 h, and the MIC was determined by visually inspecting the wells for growth of the pathogen by measuring OD_600_. The lowest concentration of the CFS at which no visible growth of the pathogen was observed was defined as the MIC (Lim et al., 2018).

The antimicrobial activity by partially purified bacteriocin, bacterial culture sample A and B, were grown in MRS broth for 24 h under shaking conditions at 37°C for 48 h. After incubation, CFS was collected by centrifugation (10,000 rpm, 15 min, 4°C). 500 ml of CFS was precipitated by 80% saturated ammonium sulfate. After overnight stirring at 4°C, the resulting precipitate was collected by centrifugation at 12,000 × *g* for 15 min and dissolved in a minimal volume of 0.1 M sodium acetate buffer (pH 6.5), which was further centrifuged (12,000 × *g* for 15 min) and the supernatant was collected. The collected supernatant was used to study the antibacterial activities of the samples that were tested against pathogenic Gram-positive and Gram-negative bacteria. The indicator strains were inoculated in the appropriate soft agar media, and the antibacterial activities were determined by measuring the zone as previously described. CFS with neutralized pH 7.0 was used to study antimicrobial activity against pathogenic bacteria. All experiments were conducted in triplicate. In order to investigate the acid production properties of the Lactobacilli strains, MRS medium containing 0.017% W/V bromocresol purple was utilized. Bromocresol purple functions as a pH indicator by changing its color from purple to yellow under acidic conditions.

#### 2.1.5. Hemolytic assay and antibiotic susceptibility

The hemolytic activities were assessed using the cells streaked on Columbia agar plates supplemented with 5% defibrinated buffalo blood and cultured for 48 h at 37°C to determine their hemolytic activity. The development of a visible zone of hemolysis encircling the colonies (++hemolysis), partial hemolysis, as well as a greenish-brown zone (+hemolysis) or no reaction (+hemolysis) was used to evaluate the hemolytic reaction after incubation. *S. aureus* ATCC 25923 cells were used as positive controls. Furthermore, potential probiotic bacterial strains were tested for their susceptibility to antibiotics using the disc diffusion method according to the criteria of the National Committee for Clinical Laboratory Standards with some modifications. HiMedia 20 Icosa Universal-1, Icosa Universal-2, and Icosa G-I-Plus antibiotic discs containing a total of 20 different antibiotics were placed on the surface of the solid nutrient agar, and after incubation for 24 h at 37°C, the diameters of the inhibition zones were noted.

#### 2.1.6. Fatty acid methyl esterase analysis and exo-polysaccharide secretion identification

The fatty acid methyl esterase (FAME) analysis was conducted using a comprehensive strategy that involved several steps ranging from sample preparation to GC-MS operation and analysis. The objective of the analysis was to determine the change in fatty acid components of two different bacterial cells. To achieve this, two sets of samples were prepared: the first set involved harvesting cells from MRS agar plates, while the second set involved collecting cell pellets after centrifugation of young active cultures. The samples were then subjected to the FAME analysis using the aforementioned strategy. The peaks were compared with standard esters using gas chromatography fatty acid methyl esters (GC-FAME) method as described and reported by Sasser (2006) and Das et al. (2019). From the supernatant of the culture, metabolites extraction and derivatization were performed. Here protein precipitation was done using ACN (acetonitrile) and allowed to dry overnight at 65–70°C, followed by derivatization using N,O-Bis(trimethylsilyl)trifluoroacetamide (BSTFA) was performed at 70°C. Afterward, GC-MS run was performed to separate and analyze the samples (Kinani et al., 2008). Exo-polysaccharide (EPS) secretion was detected by growing culture on MRS medium and also supplementing MRS media with 2% sugar concentrations of monosaccharides such as glucose, fructose, and galactose (Nambiar et al., 2018; Butorac et al., 2021). Colonies which were obtained after streaking followed by incubation for 24 h, were observed and analyzed using stereo microscopy (Magnüs MSZ-Bi, Olympus Pvt. Ltd., Noida, India).

#### 2.1.7. Cell adhesion assay

To verify the cell adhesion assay, the probiotic cells were labeled with CFDA-SE and analyzed by flow cytometry after adhering to epithelial cells. Entire protocol was performed following previously described protocol in Wang et al. (2005) and Zhang et al. (2022), with slight modifications. Probiotic cells were harvested from overnight grown culture at 3,000 × *g* for 10 min. To remove excess media, the cell pellet was washed twice with sterile PBS. Here, the probiotic cells were labeled with CFDA-SE (50 μM) at 37°C for 20 min. The labeling reaction was terminated once the incubation period was accomplished by pelleting the cells followed by washing with sterile PBS twice, to remove excess dye molecules as described in Wang et al. (2005). As described in Zhang et al. (2022), by doing certain modifications, goat endometrial cells were seeded in the 24 well plate at a concentration of 50,000 cells/well and incubated at 37°C in 5% CO_2_. This was followed by 100 μl aliquot of labeled probiotic cells (10^8^ cells/ml in RPMI-1640 medium) were then added to a 24-well plate in RPMI medium and incubated at 37°C in 5% CO_2_ for 2 h. Supernatant was aspirated and cells were washed twice with 500 μl of sterile PBS. Later, the cells were treated with 0.05% Triton X-100 and incubation of the system was carried out for 10 min. After which the sample was analyzed in Flow Cytometer (BD FACSAria Fusion™).

### 2.2. *In silico* genome analysis of two lactobacillus bacteria

#### 2.2.1. DNA extraction and genome sequencing

Bacterial cultures were grown in MRS broth medium for 24 h at 37°C in the shaker incubator. After 24 h of incubation, the overnight grown culture was centrifuged at 5,000 × *g* for 5 min to obtain bacterial cell pellets. For DNA extraction Qiamp DNA mini kit was used, following the lysozyme (20 mg/ml) treatment that was given for 1 h at 37°C in shaking conditions initially and then followed by manufacturer’s instructions. The DNA quality (260/280 and 260/230 ratio) was determined using the QIAexpert system (QIAGEN, Germany) as well as with electrophoresis on a 1% agarose gel. DNA quantification was performed using the Qubit dsDNA HS Assay Kit using Qubit 4.0 (Thermo Fisher Scientific, MA, USA). Prior to whole-genome sequencing, DNA from bacterial cultures were amplified using Thermal Cycler (Veriti, Applied Biosystem) and identified using 16S rRNA gene sequencing, using 3500xL Genetic analyzer (Applied Biosystems). The Ion Plus Fragment Library Kit (Thermo Fisher Scientific, MA, USA) was used to prepare the genomic library and the quality of the library was assessed using Bioanalyzer (Agilent) and the Qubit Fluorometer was used to quantify library concentration (Thermo Fisher Scientific). Emulsion PCR of the finally diluted library (8 pM) was carried out using the Ion OneTouch 2 system. Whole genome sequencing was performed on the Ion GeneStudio™ S5 System using a 520 chip and 400 bp sequencing chemistry.

#### 2.2.2. *De novo* genome assembly and genome annotations

The raw reads of both samples were first error corrected with software PRINSEQ 0.20.4, reads with an average quality score BELOW 50 AND length <50 bases were discarded, and then trimmed read’s quality were checked by the FASTQC program. Software SPAdes (Bankevich et al., 2012) was used with an optimum k-mer length of 127 for constructing genome assembly, hereafter the completeness of the assemblies for both samples was assessed with QUAST 5.0.2 (Mikheenko et al., 2018). The completeness of the meta-assemblies, one for each of sample A and B, was assessed with the software BUSCO (Manni et al., 2021) using the Alveolata dataset of BUSCOs. Genome annotation was done using Prokka version v1.11 (Seemann, 2014). Aragorn (Laslett and Canback, 2004) for tRNA prediction, RNAmmer (Lagesen et al., 2007) for rRNA prediction, Infernal (Kalvari et al., 2018) for ncRNA prediction, and Prodigal (Hyatt et al., 2010) for protein coding sequence prediction, respectively. The web server EggNOG v5.0^1^ was used for a cluster of orthologous genes (COG) analysis (Huerta-Cepas et al., 2019). Furthermore, the genomes of sample A and B were studied. The presence of putative virulence genes using the Virulence Factor of Bacterial Pathogen Database (VFDB) (Chen et al., 2005) to identify the presence of putative virulence genes. The presence of CRISPR repeats was predicted using the CRISPRFinder tools (Grissa et al., 2008).

#### 2.2.3. Comparative genome analysis

Whole genome sequences of *Lactiplantibacillus plantarum* and *Pediococcus pentosaceus* from animal origin were retrieved from NCBI genomes. The NCBI details of the genomes used for comparison can be found in Supplementary Table 4. All the genomes was annotated with Prokka version v1.11 (Seemann, 2014). Comparative pan genome analysis of 8 genomes of *P. pentosaceus* and 24 genomes of *L. plantarum* was done using anvio’s pan-genome workflow (Eren et al., 2015). This workflow consists of three main steps: the first one included the generation of anvio’s genomes storage using the program “Anvi-gen-genomes-storage” to store DNA from external genomes. The second step consisted of the generation of an Anvi’o pan database using the program “anvi-pan-genome” to run the pangenomic analysis and finally the distribution visualization of gene clusters across genomes. This performs GC content, redundancy, genome completion, number of gene clusters, ANI studies, phylogeny based on average nucleotide identity distance-based, core genes, and single-copy genes from all the genomes. Phylogeny based on the 16S rRNA gene was also studied, where a tree was inferred with the FastME 2.1.6.1 software. It has inferred minimum evolution tree with branch support (Lefort et al., 2015). AMR genes from the genomes were identified using the Comprehensive Antibiotic Resistance Database (CARD) (McArthur et al., 2013). Secondary metabolite biosynthetic gene clusters are predicted by antiSMASH 5.0 from the genome (Blin et al., 2019). DBCAN Meta server^2^ was used for CAZy annotation whereas HMMER is used for annotation of CAZyme domain boundaries. CAZyme domain HMM database includes glycoside hydrolases (GHs), glycosyltransferases (GTs), carbohydrate esterases (CEs), carbohydrate-binding enzymes (CBM), auxiliary active enzymes (AAs), and polysaccharide lyases (PLs) were among the enzymes in the database (Zhang et al., 2018). Bacteriocin identification and comparison were performed using BAGEL 4 (de Jong et al., 2006). Similarly secondary metabolite gene region cluster were identified with antiSMASH 6.0 (Blin et al., 2021).

### 2.3. *In vivo* experiment

For the *in vivo* experiment, buffaloes were enrolled at ambulatory clinics run by the Kamdhenu University in Village Sanoda, Tehsil Dehgam, District Gandhinagar. Farmers from Village Sanoda and nearby villages visited the clinic for the treatment of their animals. During October 2021 to January 2022, 115 buffaloes, age ranged from 4.5 to 12 years (4.6 ± 2.9 years; median ± SD years) with bodyweight from 300 to 500 kg (349 ± 90 kg; median ± SD kg) diagnosed with clinical endometritis were enrolled. Confirmation of the diagnosis was carried out using rectal palpation, history, and cervico-vaginal discharge (CVM) scoring (0–5). Cervical vaginal discharge scoring was conducted in accordance with the protocol described in Sheldon et al. (2010). The demographic data, such as lactation, parity, days postpartum, first estrus date after calving, CVM scoring, owner’s information, and whether practicing AI or natural service, were recorded.

Owing to a lack of information (18) and the sale of animals (5), these buffaloes were excluded from the experiment. Buffaloes were randomly allocated into three treatment groups: Group A (*n* = 40), receiving intra-vaginal administration of 40 × 10^8^ colony forming unit (CFU)/ml of *L. plantarum* KUGBRC (LPKUGBRC); Group B (*n* = 23), receiving intra-vaginal administration of 40 × 10^8^ CFU/ml of *P. pentosaceus* GBRCKU (PPGBRCKU); and Group C (*n* = 29), receiving intra-vaginal administration of a placebo treatment of Normal saline (NS) at a volume of 40 ml. On a weekly basis (specifically on days 7, 14, and 21), the farmers were contacted to examine the reproductive health of the buffaloes. If a buffalo exhibited estrus signs with clear CVM discharge (CVM score 0), AI or natural service was carried out. After AI/natural service, a trained veterinarian evaluated the pregnancy status of the buffaloes on day 60 via rectal palpation.

### 2.4. Statistical analysis

For *in vitro* analysis, measurements are provided as mean ± SD for all experiments, which were carried out in triplicate. Statistical significance of the data was estimated by applying *t*-test using Microsoft Excel^®^. *p*-Values ≤ 0.05 were considered to be statistically significant (Joshi et al., 2019). For *in vivo* analysis, all data were recorded in Excel spreadsheets. All analysis was performed using SPSS software (SPSS 26.0; IBM India Pvt. Ltd., Bengaluru, India). Descriptive statistical function was used to evaluate descriptive values (mean, median, SD, and SE) and frequencies. Effect of three different treatments on days between treatment intervention to estrus induction was evaluated using univariate analysis while effect of three treatments on CVM score on day 0, 7, and 14 or first estrus day. Effect of parity and months were evaluated keeping them as a covariate in the model and found non-significant, hence, removed from the analysis. *p*-Values ≤ 0.05 were considered to be statistically significant. The effect of three treatments on pregnancy rate was evaluated using Chi-square analysis.

## 3. Results and discussion

### 3.1. Phenotypic characterization of potential isolates

**FIGURE 1:**
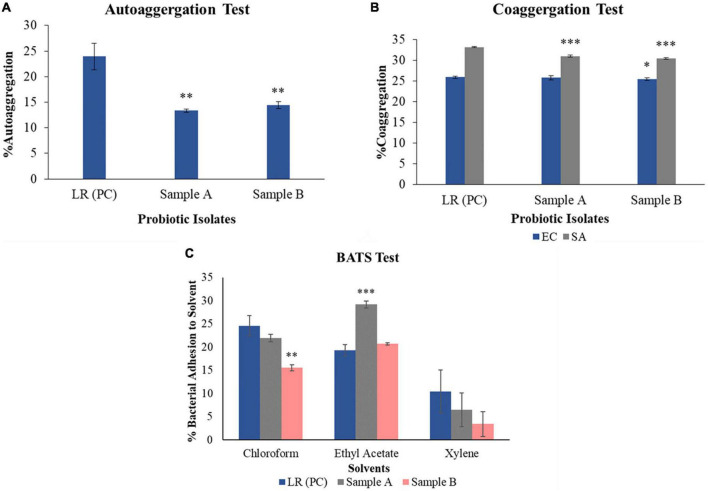
*In vitro* analysis of obtained isolates showing characteristics to be probiotic. **(A)** Auto-aggregation ability of each probiotic sample, sample A and sample B are less as compared to positive control – *Lacticaseibacillus rhamnosus* GG by 43.76 ± 0.28 and 39.27 ± 0.67%, respectively. **(B)** Co-aggregation capacity of sample A with pathogenic *E. coli* and *S. aureus* is found to be lower by 0.43 ± 0.52 and 6.68 ± 0.26%, while sample B shows less activity by 1.52 ± 0.31 and 8.33 ± 0.16% with *E. coli* and *S. aureus*, respectively, as compared to positive control – *Lacticaseibacillus rhamnosus* GG. **(C)** BATS assay which shows the activity of each solvent, i.e., chloroform, ethyl acetate, and xylene against sample A and sample B. When both the samples are treated with chloroform, sample A showed 10.24 ± 0.79% while sample B showed 36.42 ± 0.68% less activity as compared to the positive control – *Lacticaseibacillus rhamnosus* GG. When both the samples were treated with ethyl acetate, sample A showed 51.36 ± 0.74% and sample B showed 7.36 ± 0.22% more activity, as compared to the positive control – *Lacticaseibacillus rhamnosus* GG. On treating sample A with xylene, it showed as 30.68 ± 3.61%, while sample B showed 63.18 ± 2.67% less activity as compared to positive control – *Lacticaseibacillus rhamnosus* GG. Three replicates are included for each of the experimental as well as control sets. **(A)** *R*^2^ = 0.66; **(B)** *R*^2^ (EC) = 0.94 and *R*^2^ (SA) = 0.89; **(C)** *R*^2^ (sample A) = 0.44 and *R*^2^ (sample B) = 0.46. *p < 0.05, ^**^p < 0.01, ^***^p < 0.001.

Vaginal swabs were collected from the healthy buffaloes of which a total 709 primary bacterial isolates with putative probiotic activity were studied. Among all the isolates, two bacterial cultures were found to exhibit *in vitro* potential probiotic characteristics, which was further processed to study *in silico* activity. Sample A and B were able to grow on MRS agar, and showed white globular smooth colonies, sample B showed similar type of colony characteristics, however, smaller in size. And, they were identified as Gram-positive, medium-size roads in the chain and cocci, respectively, as shown in Supplementary Figure 2. The catalase test, which validates the presence of catalase for antioxidant properties to break H_2_O_2_, is showing negative results for both samples. Both bacteria showed yellow color zones surrounding colonies on MRS agar plates supplemented with bromocresol purple, which suggest that these bacteria have acid production capacity, as shown in Supplementary Figure 1. The result of the auto-aggregation test for bacterial strain sample A and B showed 43.76 ± 0.28 and 39.27 ± 0.67% lower activity respectively as shown in Figure 1, when compared with known probiotic LRGG. The ability of probiotic bacteria to aggregate necessary for probiotic function. This would allow one to attach and compete with the pathogenic bacteria that exist on the epithelial cell lining of infectious uterine lining of bovines. Capacity of bacteria to form cellular aggregates can contribute to the persistence at the site of administration (Carmen and Jussi, 2008).

Two lactobacilli strains sample A and B were tested for the co-aggregation after incubation with pathogenic strains *E. coli* and *S. aureus*. Sample A and B showed 0.43 ± 0.52 and 1.52 ± 0.31% less activity against *E. coli*, while with *S. aureus*, sample A and B showed 6.68 ± 0.26 and 8.33 ± 0.16% reduction in activity respectively as compared to positive control – LRGG. This activity explains their ability to compete in a pathogenic environment. Co-aggregation of probiotic bacteria with pathogens establishes a hostile environment for the pathogens, reflecting lowered pathogen growth, easier pathogen clearance, and re-establishment of indigenous microbiota (Younes et al., 2012). The BATS assay was performed using three different solvents, namely, chloroform, ethyl acetate, and xylene. When both the samples were treated with chloroform, sample A showed 10.24 ± 0.79% while sample B showed 36.42 ± 0.68% lower activity as compared to the positive control. Afterward, on treating both the strains with ethyl acetate, sample A showed 51.36 ± 0.74% and sample B showed 7.36 ± 0.22% more activity as compared to the positive control. Also, on treating sample A cells with xylene, it showed values like 30.68 ± 3.61%, while with sample B it showed 63.18 ± 2.67% less activity as compared to the positive control. This data is graphically shown in Figure 1. This test is used to evaluate the hydrophobic/hydrophilic nature of the cell surface properties experimentally using solvents like xylene, chloroform, and ethyl acetate (Kos et al., 2003). These solvents are attributed to the carboxylic acid group and Lewis acid-base interaction to the cell surface by showing electron donor (basic) and electron acceptor (acidic) properties. The hydrophobic and hydrophilic ability is being defined from proteins and polysaccharides present on the bacterial cell surface (Xu et al., 2009). Xylene being apolar solvent, helps in determining cell surface hydrophobicity while chloroform is a polar acidic solvent, which determines electron donor capacity and ethyl acetate being polar basic solvent, exerts electron acceptor properties of the bacterial cell surface (Xu et al., 2009). Inferring from the obtained results, sample A and B showed lower activity in xylene and chloroform and higher activity in ethyl acetate, as compared to positive control. The data obtained from the experiment reveals that sample A and B exhibit varying degrees of cell surface hydrophobicity and electron acceptor or donor properties when compared to the positive control. Specifically, sample A demonstrated higher activity with all three solvents (chloroform, ethyl acetate, and xylene) as compared to sample B. These results suggest that both samples have the capacity to adhere to surfaces, but sample A expressed this ability to a greater extent *in vitro* compared to sample B.

### 3.2. Antimicrobial activity and susceptibility to antibiotics

Cell-free supernatant from sample A and B showed distinct clear zones against pathogens, *E. coli* and *S. aureus* as shown in Supplementary Figure 4A. In addition, partially purified bacteriocin from cell-free supernatants of sample A and B with 80% ammonium sulfate precipitation also showed an inhibitory effect on *E. coli* and *S. aureus*, as shown in Supplementary Figure 4B. However, there were no zones observed in neutralized CFS when studied against pathogenic bacteria. In the study, neutralized CFS did not exhibit any inhibitory activity against pathogenic bacteria. Moreover, when partially purified bacteriocin was compared to concentrated CFS, the latter displayed greater inhibitory efficacy, as shown in Table 1. It is worth noting that the absence of inhibitory activity in neutralized CFS may be due to the pH-neutralization process, which can negatively affect the stability and functionality of the bacteriocin. These findings suggest that the bacteriocin in its native, concentrated CFS form may have a greater potential as an antimicrobial agent against pathogenic bacteria. This also signifies the antagonistic effect of other metabolites that confers the inhibition against pathogens.

**TABLE 1:** Inhibitory activity of cell-free supernatant (CFS) and partially purified bacteriocin from ammonium sulfate precipitation of sample A and B against pathogens by potential probiotics and by positive control *Lacticaseibacillus rhamnosus* GG (LRGG).

Pathogen	Product	Sample A	Sample B	LRGG
*E. coli*	CFS	24 ± 1	26 ± 1.5	23 ± 1
	Bacteriocin	19 ± 1	17 ± 1	–
*S. aureus*	CFS	23 ± 0	23 ± 0.5	22 ± 0.5
	Bacteriocin	16 ± 1	21 ± 1	–

**FIGURE 2:**
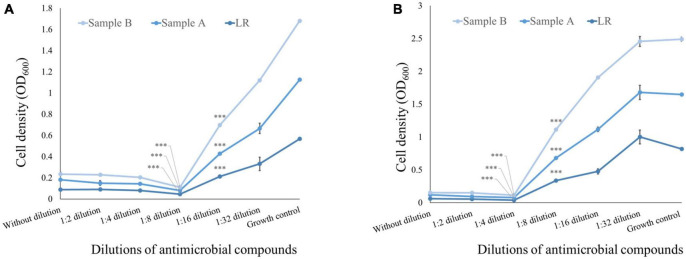
Minimum inhibitory concentration of antimicrobial compound from the 48 h CFS of grown culture against pathogenic bacteria. **(A)** MIC of CFS against *E. coli* obtained from positive control LRGG between 1:8 and 1:16 dilution is found to be 91.95*** ± 0.0005 and 62.55*** ± 0.0035%, respectively; while the MIC of sample A and sample B between 1:8 and 1:16 was found to be 93.62*** ± 0.001 and 61.30*** ± 0.007% for sample A, while the MIC of sample B was found to be 94.57*** ± 0.003 and 51.66*** ± 0.006%. Here, growth control shows presence of *E. coli* in growth medium. **(B)** MIC of CFS against *S. aureus* obtained from positive control LRGG between 1:4 and 1:8 dilution is found to be 95.05*** ± 0.01 and 58.73*** ± 0.0055%, respectively; while the MIC of sample A and sample B between 1:4 and 1:8 was found to be with 95.26*** ± 0.0033 and 58.51*** ± 0.001% for sample A, while the MIC of sample B was found to be 95.77*** ± 0.0025 and 48.48*** ± 0.004%. Here, growth control shows presence of *S. aureus* in growth medium. The entire experiment was conducted in triplicates. **(A)** *R*^2^ = 0.99 (sample A) and *R*^2^ = 0.97 (sample B); **(B)** *R*^2^ = 0.86 (sample A) and *R*^2^ = 0.83 (sample B). ^***^p < 0.001.

To determine the MIC of CFS, a broth dilution method was employed. MIC is defined as the lowest concentration of an antibiotic that can completely inhibit visible growth of a microorganism under specific *in vitro* conditions. Thus, the broth dilution method was used to quantify the MIC of CFS against the pathogens of interest in the study. As mentioned in Figure 2, the MIC of CFS was evaluated against *E. coli* using the positive control LRGG. The MIC for LRGG was found to be between 1:8 and 1:16 dilution, with a corresponding inhibition percentage of 91.95 ± 0.0005 and 62.55 ± 0.0035%, respectively. Similarly, for sample A and B, the MIC was observed between 1:8 and 1:16 dilution. The inhibition percentages for sample A were 93.62 ± 0.001 and 61.30 ± 0.007%, while for sample B, they were 94.57 ± 0.003 and 51.66 ± 0.006%, respectively. These results suggest that both samples A and B have comparable inhibitory activity against *E. coli* as compared to the positive control LRGG, at dilutions ranging from 1:8 to 1:16. The MIC of CFS was determined against *S. aureus* using the positive control LRGG. The MIC for LRGG was found to be between 1:4 and 1:8 dilution, with a corresponding inhibition percentage of 95.05 ± 0.01 and 58.73 ± 0.0055%, respectively. Similarly, for sample A and B, the MIC was observed between 1:4 and 1:8 dilution. The inhibition percentages for sample A were 95.26 ± 0.0033 and 58.51 ± 0.001%, while for sample B, they were 95.77 ± 0.0025 and 48.48 ± 0.004%, respectively. It is noteworthy that the entire experiment was conducted in triplicates, as mentioned in Figure 2. These results suggest that both samples A and B have comparable inhibitory activity against *S. aureus* as compared to the positive control LRGG, at dilutions ranging from 1:4 to 1:8.

Antimicrobial activity of any bacteria is a significant characteristic for showing competitive inhibition against pathogenic infections which involves secretions like low molecular weight proteins known as bacteriocins and/or bacteriocin-like inhibitory substances (BLIS), short chain fatty acids, organic acids, or hydrogen peroxide (Srinivasan et al., 2013; Gaspar et al., 2018; Zommiti et al., 2018). Antibiotic susceptibility of sample A showed sensitivity toward beta lactam class of drugs, nitrofuran, cephalosporins, and phenicol antibiotics, while sample B showed sensitivity against beta lactam, cephalosporins, macrolides, lincomycin, phenicol antibiotics, and tetracycline class of drugs.

### 3.3. FAME analysis and EPS secretion

**FIGURE 3:**
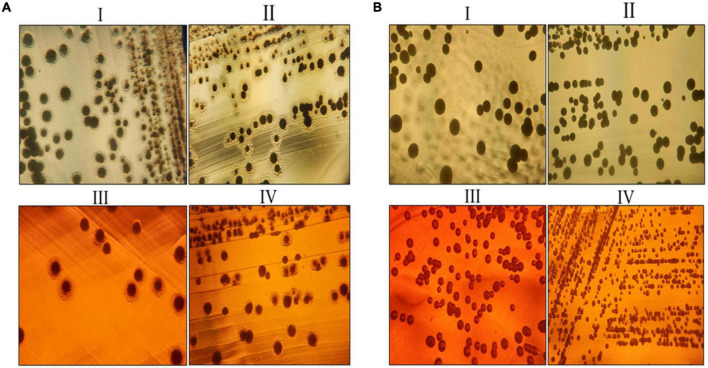
Exo-polysaccharide secretion around the colony of sample A and sample B. **(A)** EPS secretion around the colony of sample A. **(B)** EPS secretion around the colony of sample B. Where, (I) MRS medium, (II) MRS medium supplemented with 2% galactose, (III) MRS medium supplemented with 2% fructose, and (IV) MRS medium supplemented with 2% glucose.

In sample A and B, the number of fatty acids detected are as shown in Table 2. The genes responsible for fatty acid synthesis are studied *in silico* where a number of genes were identified from the KEGG pathway. There are few fatty acids of which no genes were identified from the KEGG database. trans-13-Octadecenoic acid, methyl ester, pentadecanoic acid, 14- methyl-, methyl ester, phthalic acid, butyl oct-3-yl ester, n-hexadecanoic acid, and 10-nonadecenoic acid, methyl ester was common in sample A and B, out of which trans-13-octadecenoic acid, methyl ester showed presence in KEGG database. Few fatty acids were only found in sample A and B. Out of which sample A showed octacosyl trifluoroacetate, 2-hexadecenoic acid, methyl ester, (E)-, methyl stearate, 1-dodecanol, 2- octyl-, acetate, 10-octadecenoic acid, methyl ester and trans-13-octadecenoic acid, methyl ester was obtained after doing FAME analysis and genes were found *in silico*. Sample B showed presence of 1,2-benzenedicarboxylic acid, butyl octyl ester and carbonic acid, eicosyl vinyl ester *in silico* and also by doing FAME analysis. Gene associated with EPS secretion was found in both the probiotic isolates as manifested in Table 3. Gene of polysaccharide polymerase was found in sample A, while sample B showed absence of wzy gene. This gene is responsible for polymerizing monomers with the help of polymerase wzy (Butorac et al., 2021). Here, sample A shows secretion surrounding the cell colony in all media plates, which were supplemented with different monosaccharide sugars, when observed microscopically; as shown in Figure 3.

**TABLE 2:** Fatty acid methyl esters analysis and *in silico* gene finding for fatty acid metabolites from KEGG database.

Bacterial sample	Fatty acids detected	KO IDs from KEGG pathway	Activity	References
Sample A	Phthalic acid, butyl undecyl ester	No gene found	Anti-microbial activity, anti-fungal activity, anti-inflammatory activity	Huang et al., 2021
	Octacosyl trifluoroacetate	K00665, K00668, K11533, K00208, K2371, K10780, K00209, K07512, R04958, R04959	Anti-microbial activity, anti-oxidative activity	Tyagi et al., 2021
	2-Hexadecenoic acid, methyl ester, (E)-	K00665, K00668, K11533, K00208, K371, K10780, K00209, K07512, R04969, R04970	Anti-microbial activity	Shaaban et al., 2021
	Methyl stearate	K111533, K00208, K02371, K10258, R07765	Anti-inflammatory, lipid metabolism regulator, anti-helminthic	Adnan et al., 2019
	1-Dodecanol, 2- octyl-, acetate	K00665, K00668, K11533, K00208, K02371, K10780, K00209, K07512, R04724, R04725	Anti-microbial activity	İnci et al., 2022
	10-Octadecenoic acid, methyl ester	K00665, K00668, K11533, K00208, K2371, K10780, K00209, K07512, R04958, R04959	Anti-microbial activity, anti-fungal activity	Rahman et al., 2014; Serra et al., 2020
Sample A and sample B	Trans-13-Octadecenoic acid, methyl ester	K11533, K02372, K10703, R07764	Anti-inflammatory activity	Taylor and Pillarisetty, 2023
	Pentadecanoic acid, 14- methyl-, methyl ester	No gene found	Defense against microbial invasion, anti-carcinogenic activity	Nadathur et al., 1996; Manilal et al., 2009
	Phthalic acid, butyl oct-3-yl ester	No gene found	Anti-microbial activity, insecticidal activity	Huang et al., 2021
	n-Hexadecanoic acid	No gene found	Anti-bacterial activity, anti-fungal activity, anti-quorum sensing activity	Cetin et al., 2010; Waheed et al., 2019; Patel et al., 2022
	10-Nonadecenoic acid, methyl ester	No gene found	Biodiesel production, antitumor properties	Bharti et al., 2014; Allen-McFarlane et al., 2019
Sample B	Tridecanoic acid, 12- methyl-, methyl ester	No gene found	Antitumor activity, flavoring agents, surfactants, anti-fungal activity, anti-bacterial activity	Elaiyaraja and Chandramohan, 2018; Duraisamy and Raja, 2020
	Tetradecanoic acid, 12- methyl-, methyl ester	No gene found	Anti-fungal activity, antioxidant, cancer preventive, nematicide	Mujeeb et al., 2014
	1,2-Benzenedicarboxylic acid, butyl octyl ester	K00208	Anti-bacterial activity, anti-fungal activity	Waheed et al., 2019
	Hexatriacontyl pentafluoropropionate	No gene found	Anti-bacterial activity	Alanazi et al., 2022
	(Z)-Methyl hexadec-11-enoate	No gene found	Anti-microbial activity	Alves et al., 2020
	11-Octadecenoic acid, methyl ester	No gene found	Anti-diarrheal activity and antimicrobial activity	Ojuolape Amusan, 2020
	Carbonic acid, eicosyl vinyl ester	K00208	Anti-microbial and anti-oxidative	Chikezie et al., 2019

KEGG, Kyoto Encyclopedia of Genes and Genomes; KO, KEGG Orthology.

**TABLE 3:** Genes associated with potential probiotic properties of *L. plantarum* KUGBRC and *P. pentosaceus* GBRCKU.

Category	Genes	Abbreviation	*L. plantarum* KUGBRC	*P. pentosaceus* GBRCKU
Exopolysaccharide biosynthesis	LytR-transcriptional regulator	epsA	MCL3854916.1	MCL3859315.1
	CpsD/CapB family tyrosine-protein kinase	epsC	MCL3855360.1	MCL3858257.1
	WecB/TagA/CpsF family glycosyltransferase	gt	MCL3856989.1	MCL3859201.1
	Flippase	wzx	MCL3858247.1	MCL3855466.1
	Polysaccharide polymerase	wzy	MCL3855354.1	–
Adhesion	Class A sortase	strA	MCL3856895.1	MCL3858892.1
	MucBP domain-containing protein	mucbp	MCL3857432.1	MCL3859314.1
	LPXTG cell wall anchor domain-containing protein	lp_2940	MCL3854843.1	–
	Hsp33 family molecular chaperone HslO	hsp33	MCL3856863.1	MCL3858913.1
Oxidative stress	Catalase	–	MCL3856024.1	MCL3857992.1
	Thiol peroxidase	–	MCL3856501.1	MCL3858978.1
	Thioredoxin	–	MCL3856540.1	MCL3858440.1
	Glutathione peroxidase	–	MCL3857223.1	–
	Dyp-type peroxidase	–	MCL3855919.1	–
	Pyruvate oxidase	–	MCL3855734.1	MCL3857678.1
	Hsp20/alpha crystallin family protein	–	MCL3855869.1	MCL3858821.1
	Chaperonin GroEL	–	MCL3855835.1	MCL3858391.1
	Molecular chaperone DnaK	–	MCL3855288.1	MCL3857978.1
	Molecular chaperone DnaJ	–	MCL3855287.1	MCL3857979.1
	Na^+^/H^+^ antiporter NhaC	–	MCL3855857.1	MCL3858619.1
Nitric oxide production	FMN-dependent NADH-azoreductase	–	MCL3855651.1	–
Bile salt hydrolase	Choloylglycine hydrolase	–	MCL3855998.1	–
Polyamine production	Glutamate decarboxylase	–	MCL3855910.1	–
	Ornithine carbamoyltransferase	–	MCL3856876.1	MCL3858832.1

### 3.4. Cell adhesion assay

**FIGURE 4:**
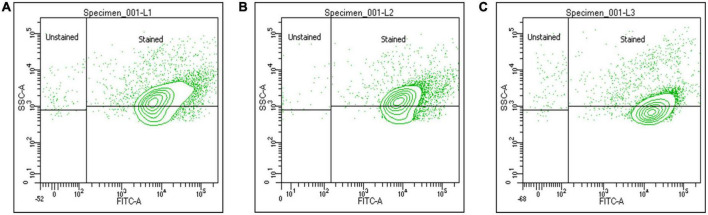
Cell adhesion assay: labeling of the probiotic bacterial cells with CFDA-SE and analyzing adherence of bacterial cells to epithelia using flow cytometer. Panel **(A)** is showing adhered *Lacticaseibacillus rhamnosus* GG cells to epithelium; panel **(B)** is showing adherence of *Lactiplantibacillus plantarum* to endometrial epithelium, while panel **(C)** is showing adhered cells of *Pediococcus pentosaceus* to epithelium. The efficiency of the CFDA-SE labeling was analyzed by measuring the fluorescence of the bacteria using FITC channel.

The approach employed to investigate the adhesion ability of sample A and sample B to epithelial cells was adapted from previously described reports (Wang et al., 2005; Zhang et al., 2022). CFDA-SE, a non-fluorescent membrane permeable ester that is converted to a fluorescent molecule by non-specific intracellular esterase and covalently linked to intracellular proteins via its succinimidyl group, was used to label the probiotic bacterial cells (Hytönen et al., 2006). Each set was allowed to capture 10,000 events and assay was performed, labeled bacteria of sample A and sample B are observed using FITC channel, as CFDA-SE dye having excitation/emission maxima ∼492/517 and its signal falls under FITC channel. The probiotic strains showed adhesion capabilities with goat endometrial cell line. Figure 4 mentions the stained probiotic cells observed after incubation and lysis of epithelial cells, showing presence of adhered probiotic cells. Unstained cells and stained unadhered cells are observed and showed in Supplementary Figure 3.

### 3.5. Bacterial identification and general genome feature

Probiotic bacterial strains studied here are confirmed by the 16S sanger sequence identification. Sample A and B were successfully sequenced with 99.86 and 98.73% identity respectively, which also showed the query covered 100 and 99%, respectively, when analyzed. Sample A was identified as *Lactiplantibacillus plantarum* KUGBRC (LPKUGBRC) and sample B as *Pediococcus pentosaceus* GBRCKU (PPGBRCKU). Genome sequencing using Ion GeneStudio™ S5 System produces data from sample A with 1,689,136 reads, and sample B reads were 4,200,647. The raw reads were first curated by discarding low-quality reads and removing adaptor sequences using the software PRINSEQ 0.20.4. Total clean reads for samples A and B were 1,665,758 and 16,591,704, respectively. These were used for further analysis. SPAdes assembler generated 77 and 22 contigs for samples A and B, respectively at k-mer 127 as showed in Table 4. Sequences of sample A and B were uploaded to the NCBI GenBank database. Further, the genome completeness of sample A and B was 98.6 and 98.2%, respectively according to the complete and single-copy BUSCOs from the BUSCO database. Using the PubMLST database, samples A and B were identified as *L. plantarum* and *P. pentosaceus* respectively, with 100% support.

**TABLE 4:** Quast based assembly statistics of whole genome sequenced sample A and B.

Assembly statistics	Contigs	Total length (BP)	N50 (BP)	GC (%)
Sample A	77	3,203,348	293,158	44.55
Sample B	22	1,797,689	380,132	37

### 3.6. Genome annotation and functional classification of probiotics

**FIGURE 5:**
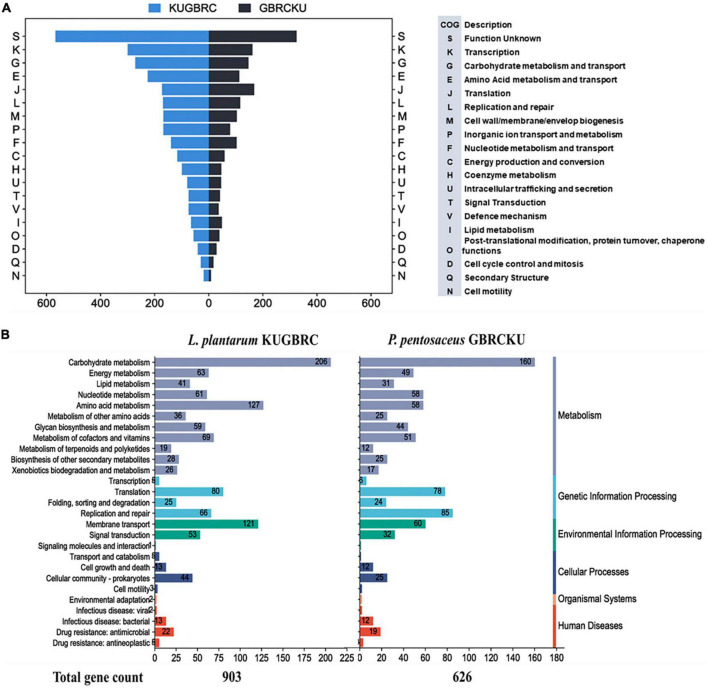
Functional annotation of *L. plantarum* KUGBRC and *P. pentosaceus* GBRCKU. **(A)** Distribution of annotated genes across the different COG functional categories of *L. plantarum* KUGBRC and *P. pentosaceus* GBRCKU. **(B)** KEGG pathway distribution of essential genes in *L. plantarum* KUGBRC and *P. pentosaceus* GBRCKU.

The complete genomes of sample A and B were annotated with NCBI PGAP (Tatusova et al., 2016), an annotation pipeline, which is specifically designated for bacterial and archaeal bacterial genomes. Protein encoding genes of sample A and B, including 2,896 and 1,711, respectively. The genomic features of LPKUGBRC and PPGBRCKU under the study are provided in Table 5. Two lactobacilli studied here harbor many probiotic-associated genes that confer benefits to the host. The reproductive ecosystem of the buffalo and cow is a diverse environment for most microorganisms. In order to survive and successfully colonize the reproductive ecosystem, probiotics LPKUGBRC and PPGBRCKU must have evolved mechanisms of adaptation. In the studied genomes, we have identified many genes encoding proteins involved in adaptation mechanisms. These stresses include temperature, osmatic stress, nitrosative stress, and oxidative stress. The detailed analysis of genes coding for those involved in the adaptive response in the genomes of LPKUGBRC and PPGBRCKU as studied in Table 3. For functional classification, the KEGG database has identified a total of 2,162 genes of strain LPKUGBRC and 1,569 genes of strain PPGBRCKU, and 2,723 genes of LPKUGBRC and 1,663 genes of PPGBRCKU has assigned with the COG database as shown in Figures 5A, B. Gene distributed across COG with the different categories revealed most abundant accounting for 76% of LPKUGBRC and 52% of PPGBRCKU of the total COG annotation. Among them, most were involved in carbohydrate metabolism and transport, amino acid transport, metabolism, and inorganic ion transport. The study acceded with relative studies where this probiotic showed versatile and flexible which can grow with multiple carbon, and nitrogen sources.

**TABLE 5:** Genomic features of *L. plantarum* KUGBRC and *P. pentosaceus* GBRCKU.

Feature	*L. plantarum* KUGBRC	*P. pentosaceus* GBRCKU
Total genome size (bp)	320,304	1,797,072
DNA G + C (%)	44.54	37
Genes (total)	3,076	1,826
CDSs (total)	2,997	1,760
Genes (coding)	2,896	1,711
CDSs (with protein)	2,896	1,711
Genes (RNA)	79	66
rRNAs	5, 7, 1 (5S, 16S, 23S)	4, 3, 2 (5S, 16S, 23S)
complete rRNAs	4, 1, 1 (5S, 16S, 23S)	4, 1, 1 (5S, 16S, 23S)
partial rRNAs	1, 6 (5S, 16S)	2, 1 (16S, 23S)
tRNAs	62	54
Pseudo genes	102	49
ncRNAs	4	3
GenBank accession	GCA_023369895.1	GCA_023369775.1

### 3.7. Phylogenetic and core genome analysis of *L. plantarum* KUGBRC and *P. pentosaceus* GBRCKU from animal origin

**FIGURE 6:**
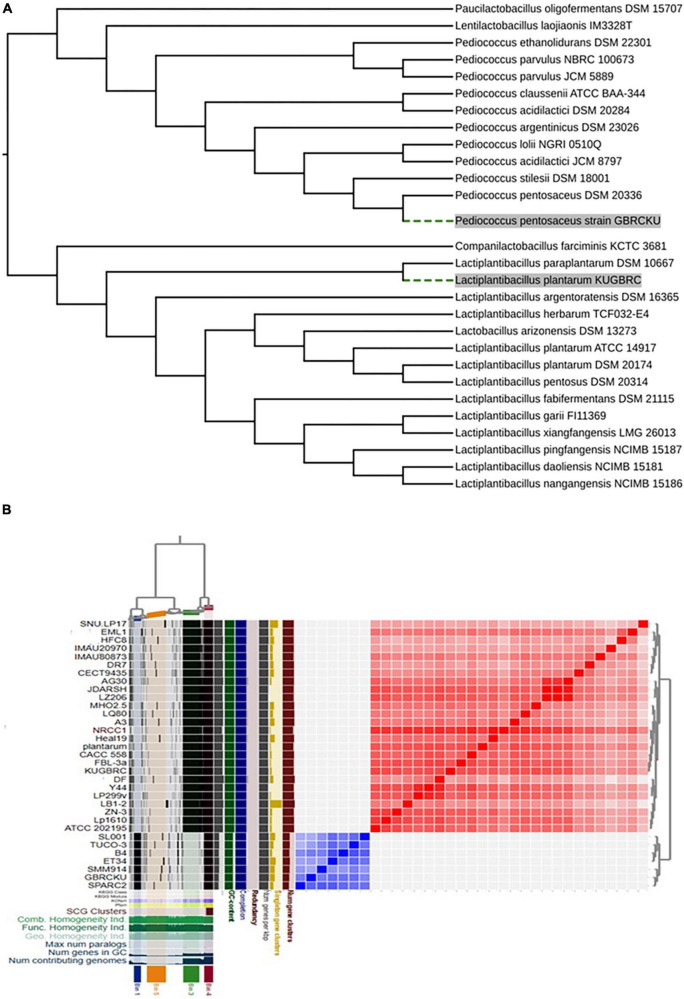
**(A)** Phylogenetic tree analysis of 16S rRNA gene sequences obtained by the neighbor-joining (NJ) showing relationship of strain *L. plantarum* KUGBRC and *P. pentosaceus* GBRCKU with related species. **(B)** Pangenome analysis clustering of genomes based on the presence/absence patterns of 23,421 pangenomic clusters. The genomes are organized in radial layers as core, unique, and accessory gene clusters (Euclidean distance; Ward linkage) which are defined by the gene tree in the center. The clades are colored based on the shared gene clusters as shown in the tree in the right top above the heatmaps and the phylogenomic groups of the strains are denoted by the corresponding colors in the pangenome tree. Heatmaps denote the functions enriched in the core-(below) and strain-specific (top) gene contents based on annotated KEGG and Pfam databases. The core-and strain-specific gene clusters are highlighted to distinguish them from dispensable genome. The figure was constructed using Anvi’o pangenomics workflow (http://merenlab.org/software/anvio/).

Comparative genome analysis of LPKUGBRC and PPGBRCKU with the closest organism isolated from the host-origin were studied. The phylogenetic tree was constructed based on 16S rRNA gene sequence where LPKUGBRC was found to be closely related to *L. plantarum* DSM 10067 and PPGBRCKU is closely related to *P. pentosaceus* DSM 20174 as shown in Figure 6A. At the whole genome level ANI values for LPKUGBRC and PPGBRCKU shares 99.31% with *L. plantarum* ZN-3 and 98.92% with *P. pentosaceus* SMM914, respectively. However, the ANI values of other strains of host-originated LPKUGBRC and PPGBRCKU share between 99.0–95 and 98.9–98.6%, respectively. For studying genomic diversity within closely related bacteria or the same species, pan-genome and core-genome methods are often used (Zhang et al., 2019). Pan-genome analysis which includes 34 genome’s phylogenetic tree analysis with a concatenated alignment of single-copy genes present in the different strains of *L. plantarum* and *P. pentosaceus*. Here we have studied the pan-genome analysis of lactobacilli genomes to highlight the novelty of the lactobacilli strains and to evaluate the probiotic capacity of the bacteria. Our pan genome analysis of lactobacilli genomes with a total of 95,712 genes resulted in 7,210 gene clusters as shown in Figure 6B. These gene clusters are further grouped in the four bins, based on their occurrence across the genome: (1) Bin 1; core P gene clusters (*n* = 4,339) from selected strains of *P. pentosaceus* genomes, (2) Bin 2; core LP gene clusters (*n* = 37,230) from selected strains of *L. plantarum* genomes, (3) Bin 3; core P + LP gene clusters (*n* = 26,083), (4) Bin 4; unique gene across the genomes (gene clusters associated with a single genome; *n* = 1,929). The unique genes and P + LP (*P. pentosaceus* + *L. plantarum*) core gene clusters corresponded to 25.39 and 10.51% of all clusters, respectively. A total of 41% of gene clusters contained genes annotated with Pfam functions. The phylogenetic position and genetic relatedness measured by ANI values and single-copy core genes define the strains. Presence of unique genes from the LPKUGBRC and PPGBRCKU strains compared with relatively closely related strains from animal origin are presented, where different strains of *L. plantarum* and *P. pentosaceus* have 33 and 85 single-copy genes, respectively. To further evaluate, the Pfam database was used to assign functions to 17 and 57 genes of the strain LPKUGBRC and PPGBRCKU’s number of unique genes, respectively. The strains LPKUGBRC and PPGBRCKU hold a unique number of distinctive genes that enable bacteria to outperform the competition for niche establishment as mentioned in Supplementary Table 2.

### 3.8. Computing bacteriocin and bioactive products from the selected genomes

**FIGURE 7:**
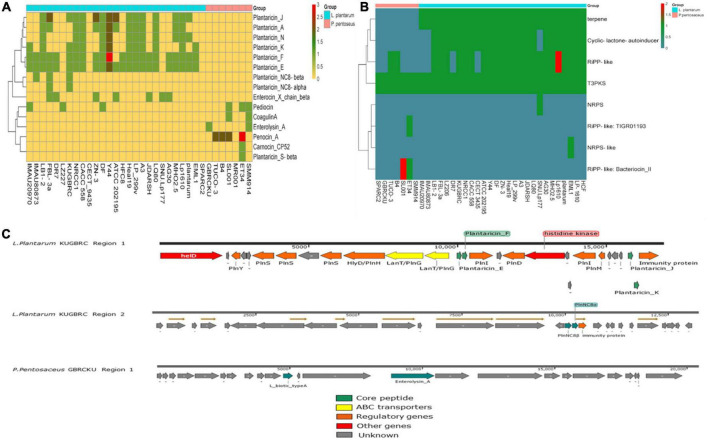
**(A)** Heatmap showing the distribution of bacteriocins in all selected strains of *L. plantarum* and *P. pentosaceus*. **(B)** Secondary metabolites comparison across all the selected strains. **(C)** Bacteriocin gene region found from the probiotic *L. plantarum* KUGBRC and *P. pentosaceus* GBRCKU.

Bacteriocin encoding genes were screened in the different selected strains of *L. plantarum* and *P. pentosaceus*, as shown in Figure 7A. The distribution of the bacteriocin encoding genes in these two different bacterial genomes are diverse and varied groups of antimicrobials that use different systems for bacteriocin modification, transport, and immunity. *In silico* analysis, showed the presence of putative bacteriocin *pln* operon in the LPKUGBRC, including class II bacteriocins, plantaricin E/F and plantaracin J/K with 100% identity, which also shows synergistic effect against target bacteria (Pal and Srivastava, 2014). Two lanT gene homologs that encode the bacteriocin ABC transporter, the ATP binding protein, and the permease protein PlnG are located upstream of the plantaricins genes. The second gene cluster shows the presence of the class II bacteriocin plantaracin NC8β/NC8α with 100% identity. In the same operon, the putative immunity protein is located downstream of the plantaracin NC8βα with 100% identity, as shown in Figure 7C. However, genes confirming the presence of bacteriocin in the PPGBRCKU include L_Biotic_type_A and Enterolysin_A in a single gene cluster, where Enterolysin_A with 40% identity and blast P gave identity 100% with peptidoglycan DD-metalloendopeptidase family protein. L_Biotic_type_A is giving 100% identity with Tail assembly protein G. *L. plantarum* is known to produce class I and II bacteriocins, including Plns A-Y, NC81F, NC8HK, and NC8βα (Wang et al., 2016). The bacteriocins produced by *L. plantarum* are known to reduce gastrointestinal diseases by inhibiting the growth of pathogens like *S. aureus* and *Listeria* (Yu et al., 2015). All plantaricins are produced as precursors with a double glycine moiety by the genes plnE and plnF, and are further exported by the PlnG and PlnH proteins (Deegan et al., 2006). For the probiotics to survive and successfully colonize the endometrium of the cattle, they must have the ability to produce secondary metabolites with antimicrobial activity to combat endometritis and metritis infections (Martin and Demain, 1980). In the studied genomes, we found the presence of gene clusters in two different probiotics potentially involved in the biosynthesis of secondary metabolites, which is shown in Figure 7B. T3pks (type III polyketide synthase), which was associated with the biosynthesis of polyketides. Polyketides are natural metabolites that comprise the basic chemical structure of various anticancer, antifungal, and anticholesteremic agents, antibiotics, parasiticides, and immunomodulators (Weissman, 2009). When secondary metabolites were compared among the different strains of LPKUGBRC and PPGBRCKU, T3pks were found to be present in all the strains of the selected genomes. Terpene is a common secondary metabolite, in addition to T3pks, in selected *L. plantarum* genomes. Terpenes play an important role in various biological processes, including membrane biosynthesis, photosynthesis, electron transport, cellular respiration, and signaling control of growth (Helfrich et al., 2019). Cyclic lactone autoinducer and Ripp-like were found to vary among *L. plantarum* genomes (Özçam et al., 2019). However, strain LPKUGBRC harbors both the gene clusters in the genome. Cyclic lactone autoinducer are cyclic peptides that play an essential role in signal transduction pathways such as quorum sensing. Gram-positive bacteria use these small peptides to measure the population density (Mull et al., 2019). Functions of Ripp-like (ribosomally synthesized and post-translationally modified peptides) are diverse in quorum sensing, acting as enzyme cofactors, roles in cellular development, mediating host-microbe interactions, but also the much sought-after antibacterial and antifungal (Kloosterman et al., 2021).

**FIGURE 8:**
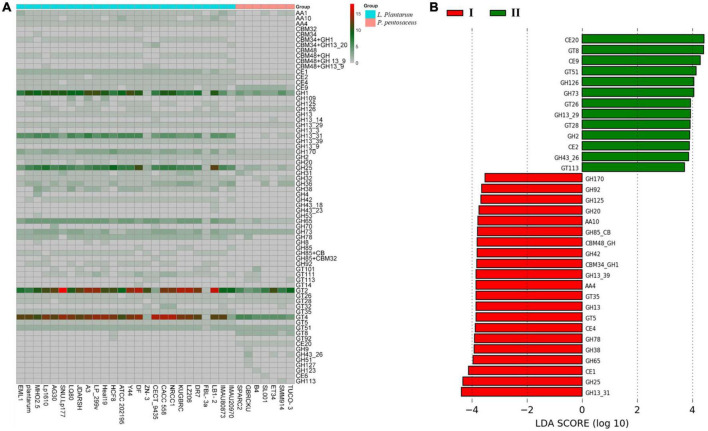
**(A)** Comparison of carbohydrate active enzyme (CaZy) with protein blast, across all the selected probiotic strains. **(B)** LeFse analysis indicates potential presence of significant different carbohydrate active enzymes; red color showing differential CaZy across (I) *L. plantarum* selected strains, and green shows significant CaZy across (II) *P. pentosaceus* selected strains.

CAZy analysis (Carbohydrate-Active enZymes) further confirmed the presence or absence of GHs, GTs, PLs, CEs, and AAs enzymes in selected genomes in Figure 8A of the study. The GH family enzyme are required for the degradation of glycosidic linkages between carbohydrates, The most abundant GH family enzymes between all strains include GH25, GH170, GH65, and GH63. The GT family mainly encodes for glycosyltransferases, where GT2 (requires for cellulose and EPS biosynthesis), GT4 (requires for sucrose synthesis), and GT51 (murein polymerase) were found to be present in all the strains. LEfSe (linear discriminant analysis (LDA) effect size) analysis to investigate significant differences in the gene families of carbohydrate-active enzymes between genomes of *L. plantarum* and *P. pentosaceus*, which were isolated from different niches. The LDA distributions of carbohydrate enzyme families and their histogram with computed LDA scores (log10) = 2 for differentially abundant enzymes between A and B are shown in Figure 8B. A description of each differentially abundant enzyme between A and B is given in Supplementary Table 1.

### 3.9. Intra-vaginal probiotic administration in buffaloes with endometritis

The study evaluated a total of 92 animals in three different groups, after applying the exclusion criteria. The probiotics groups (sample A, LPKUGBRC and sample B, PPGBRCKU) demonstrated improvement in CVM score over the period of time compared with placebo (NS) treated groups of buffaloes. However, no significant difference was observed (*p* > 0.05). The probiotic administration of LPKUGBRC has demonstrated significant reduction of on interval between treatment day to estrus induction, compared with PPGBRCKU (*p* = 0.0.2) or placebo (*p* = 0.03) treated groups, as shown in Supplementary Figure 5. Interestingly on day of estrus only five out of 40 buffaloes in LPKUGBRC group demonstrated CVM score 1, while in PPGBRCKU and NS group 10 and 2 buffaloes, respectively, demonstrated 0 CVM score out of 23 and 29 buffaloes, respectively. On day 60 after AI/natural service, the pregnancy rate was highest in the LPKUGBRC treated group (62.5%), followed by the PPGBRCKU group (52.2%) and the NS group (51.72%) treated buffaloes. However there was no significant difference observed between three groups (*p* = 0.597). The buffaloes included in this study were sourced from diverse animal farmers across various villages. As a result, factors such as farm hygiene, nutrition, and other potential variables could have impacted the outcomes of the investigation. The administration of probiotics was found to reduce the number of estrus induction days and the incidence of endometritis in buffaloes. This observed effect could be attributed to the various lactobacilli secretions that were present due to the administration of probiotics. These lactobacilli are known to have probiotic effects that help in maintaining a healthy vaginal microbiota, reducing the incidence of infections and inflammation (Genís et al., 2018; Galvão and Bicalho, 2019), and improving reproductive health. Lactobacilli are also involved in the production of lactic acid, which creates an acidic environment that is hostile to pathogenic bacteria. Therefore, the reduction in estrus induction days and endometritis in buffaloes could be due to the ability of lactobacilli to maintain a healthy vaginal microbiota and reduce inflammation, thereby improving reproductive health in buffaloes. In addition to lactic acid, bacteriocins may also play a role in the observed reduction in estrus induction days and endometritis and improvement of CVM score in buffaloes. Bacteriocins are antimicrobial peptides that are produced by lactobacilli and are known to inhibit the growth of pathogenic bacteria. These bacteriocins can selectively target and kill specific bacteria, making them a potential alternative to antibiotics in the treatment of bacterial infections. Therefore, it is possible that the reduction in endometritis in buffaloes treated with probiotics could be due to the synergistic effects of lactobacilli-produced bacteriocins and lactic acid, which work together to create an environment that is unfavorable for pathogenic bacteria. However, further research is needed to investigate the specific mechanisms by which probiotics exert their beneficial effects in buffaloes. It is important to note that probiotics have been shown to have a positive effect on the overall health and well-being of buffaloes. The administration of probiotics can improve the nutritional status of animals and enhance their immune system, making them more resistant to infections. This can be especially beneficial for buffaloes that are at risk of developing reproductive tract infections such as endometritis.

### 3.10. Safety assessment

**FIGURE 9:**
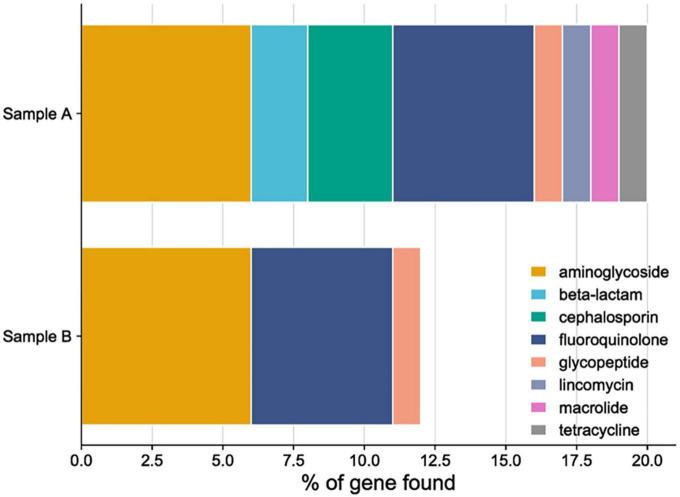
Anti-microbial resistance gene (AMR) and antibiotic resistance phenotype drug class distribution in sample A (*L. plantarum* KUGBRC) and sample B (*P. pentosaceus* GBRCKU).

The presence of antimicrobial gene resistance and their gene completeness correlated with the antibiotic sensitivity phenotype and number of genes in both of the studied probiotics, as described in Figure 9. The probable explanation for the results might be, sample A and B are originally isolated from the reproductive sites of buffaloes and cows, where exposure to pathogens and antibiotics used for treating endometritis is maximum. So, the presence of genes showing resistance to antibiotics are found. Usually, fluoroquinolones and aminoglycosides are commonly used to treat endometritis in cattle (Taylor and Pillarisetty, 2023), in both the probiotics maximum number of genes are found from each drug class. Supplementary Table 3 gives information about the genes associated with AMR and the specific drug classes that they confer resistance to. The hemolytic activity of both isolates was tested on Columbia Blood Agar plates using, where *S. aureus* acted as a positive control and LRGG as a negative control. None of the strains tested positive for α-hemolytic and β-hemolytic activity when grown on blood agar plates. Genomically, virulence genes were studied from both the genomes of probiotics, where no genes were found from the LPKUGBRC and PPGBRCKU. The strains tested exhibited negative or no hemolytic activity (Yasmin et al., 2020). Usually, probiotic strains do not show any hemolytic activities (Mangia, 2019). When it comes to safety, the absence of hemolytic enzymes is crucial, mainly during the selection of probiotic strains, when the probiotic will be used to treat any infection in the host.

## 4. Conclusion

In the present study, probiotic bacteria isolated from reproductive sites of buffaloes and cows might have the ability to combat endometritis infection caused by the pathogens. The metabolites secreted by probiotic isolates are prone to exhibit antimicrobial activity against pathogenic bacteria. The genotypic and phenotypic characteristics of LPKUGBRC and PPGBRCKU confirms their probiotic efficiency. Which indicates that these two probiotic bacteria might possess an improved ability to adapt the environment. Here we demonstrate the first report of a comprehensive analysis of LPKUGBRC and PPGBRCKU screened from the healthy uterus of bovines, commencing with the isolation and characterization and continuing via *in silico* whole genome analysis. Our research has yielded two unique strains of probiotic bacteria that demonstrate considerable potential for treating endometritis. The novelty of our probiotic lies in its origin, as we have sourced it from the healthy bovine vaginal environment, the same niche as the targeted infection. This aspect of our probiotic may confer a distinct advantage, as it may be more adept at targeting the specific microbial population associated with endometritis. We consider this finding to be of great significance for the advancement of probiotic therapies and recommend further exploration of its clinical applications.

## Data availability statement

The complete assembled genomes for *Lactiplantibacillus plantarum* KUGBRC and *Pediococcus pentosaceus* GBRCKU have been deposited in the NCBI GenBank, with accession numbers: GCA_023369895.1 and GCA_023369775.1, respectively.

## Ethics statement

This animal study was reviewed and approved by the Kamdhenu University, Gandhinagar. Written informed consent was obtained from the owners for the participation of their animals in this study.

## Author contributions

VS, MJ, and CJ designed the experimental scheme. VS, PG, BN, and KP participated in the experiment process and assisted in sampling. PG and BN completed the analysis of experimental data, the making of charts, and the initial draft. VS and MJ completed the overall modification of the manuscript. CJ and DP improved and polished the language of the manuscript. DP, MJ, and CJ provided the necessary experimental equipment and key guidance during the experiment process. All authors read and approved the final manuscript.

## Footnotes

1
http://eggnog45.embl.de

2
https://bcb.unl.edu/dbCAN2/blast.php
