# Supplementary material for: Assessing the efficacy of probiotics in augmenting bovine reproductive health: an integrated in vitro, in silico, and in vivo study
Source: Front Microbiol. 2023 May 18;14:1137611. doi: 10.3389/fmicb.2023.1137611 (PMC10232901; doi:10.3389/fmicb.2023.1137611)
Supplement: Supplementary file 1 [file Data_Sheet_1.docx]

Supplementary Material

Assessing the Efficacy of Probiotics in Augmenting Bovine Reproductive Health: An Integrated *In Vitro*, *In Silico*, and *In Vivo* Study

**Purva Gohil^1^, Bhavya Nanavati^1^, Kajal Patel^1^, Vishal Suthar^2*^, Madhvi Joshi^1*^, Deepak B Patil^2^, Chaitanya G Joshi^1^**

***Correspondence:**

1. Dr. Madhvi Joshi,

Scientist-D and Joint Director,

Gujarat Biotechnology Research Centre (GBRC), Gandhinagar,

Department of Science and Technology, Government of Gujarat, India.

Phone: +91- 99784 41233

Email: [madhvimicrobio@gmail.com](mailto:madhvimicrobio@gmail.com)

ORCID: orcid.org/0000-0003-4102-7114

1. Dr. Vishal Suthar,

Directorate of Research,

Kamdhenu University,

Sector 10 A, Gandhinagar, Gujarat, India 382010

Phone: +91-98244 91049

Email: [vsuthar28@gmail.com](mailto:vsuthar28@gmail.com)

ORCID: orcid.org/0000-0002-2136-4060

# **Supplementary Figures**


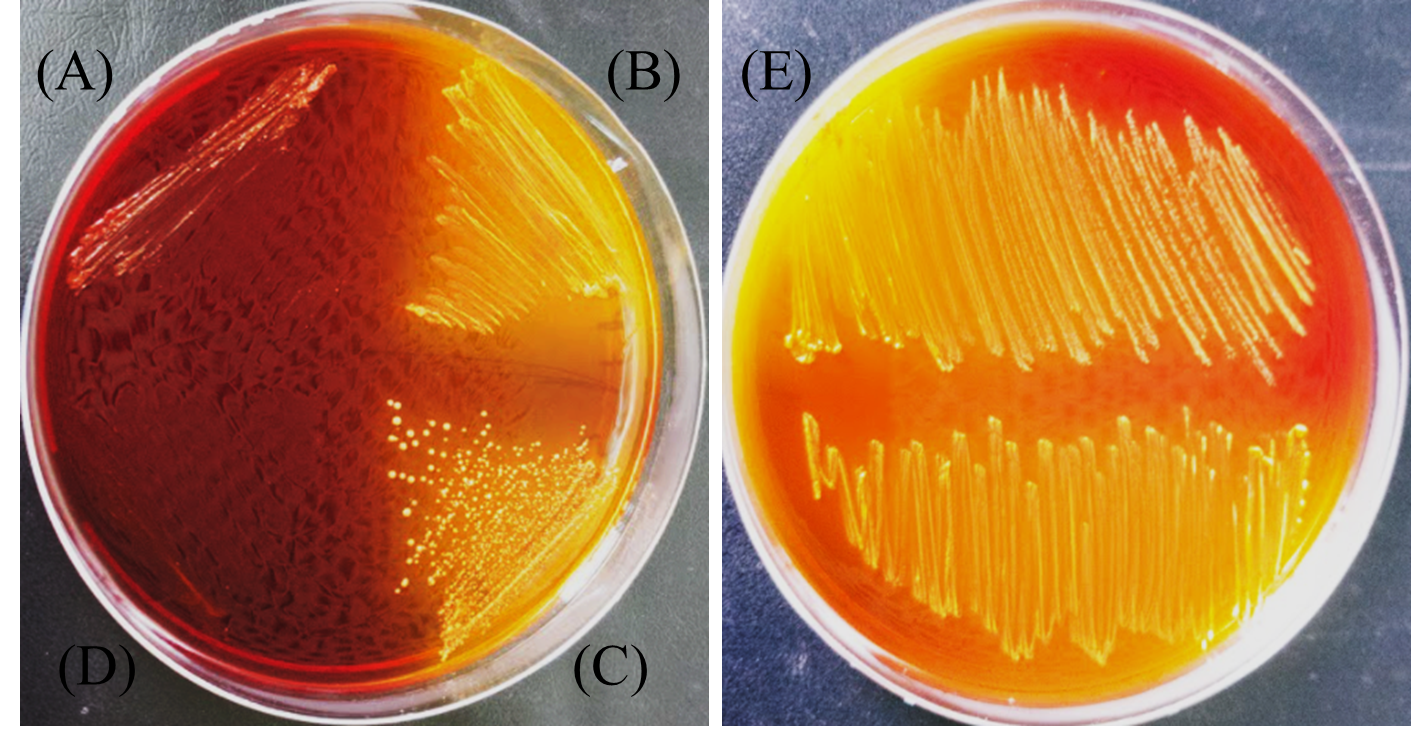


**Supplementary Figure 1.** Acid production capacity of probiotic isolates showing yellow coloration surrounding cell growth on the MRS agar plate supplemented with (0.017% W/V) bromocresol purple (A) Acid production capacity of negative control - *S.* aureus. (B) Acid production capacity of Sample A. (C) Acid production capacity of Sample B. (D) Acid production capacity of negative control - *E. coli.* (E) Acid production capacity of positive control - *Lactobacillus rhamnosus*.


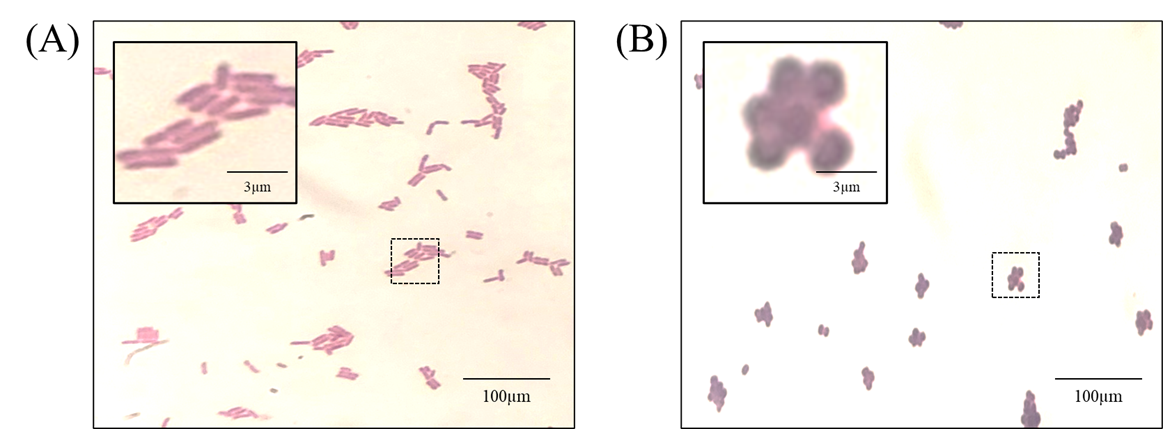


**Supplementary Figure 2.** Gram’s staining of Sample A and B under light microscope with magnification of 100x, using oil immersion lens. (A) Sample A – Gram positive rods and (B) Sample B – Gram positive cocci.


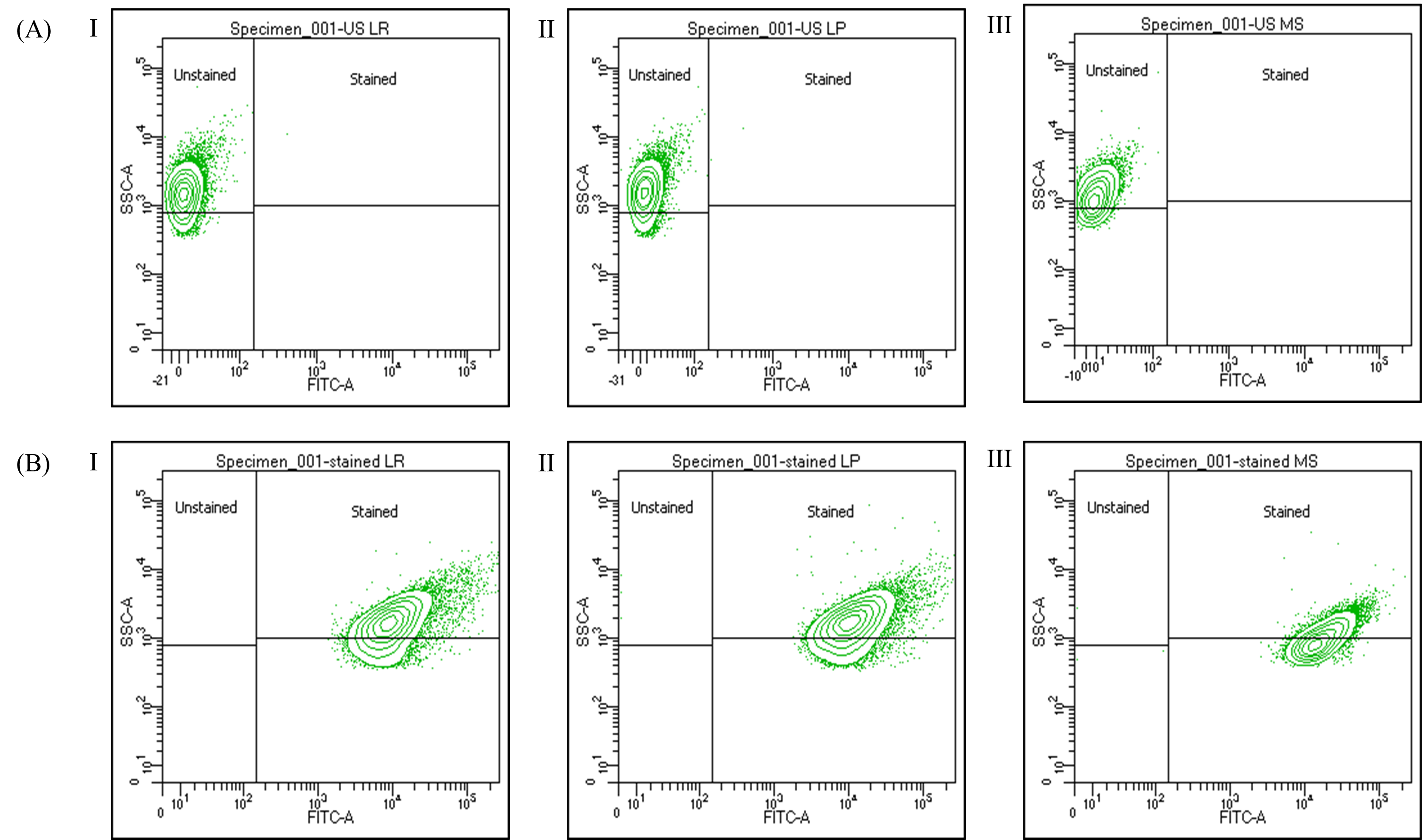


**Supplementary Figure 3.** Cell Adhesion Assay:

Labelling of the probiotic bacterial cells with CFDA-SE and analyzing (A) Unstained cells as well as (B) Stained cells

1. is showing *Lactobacillus rhamnosus* cells
2. is showing *Lactiplantibacillus plantarum* cells, while
3. is showing *Pediococcus pentosaceus* cells

The efficiency of the CFDA-SE labelling was analyzed by measuring the fluorescence of the bacteria using FITC channel.


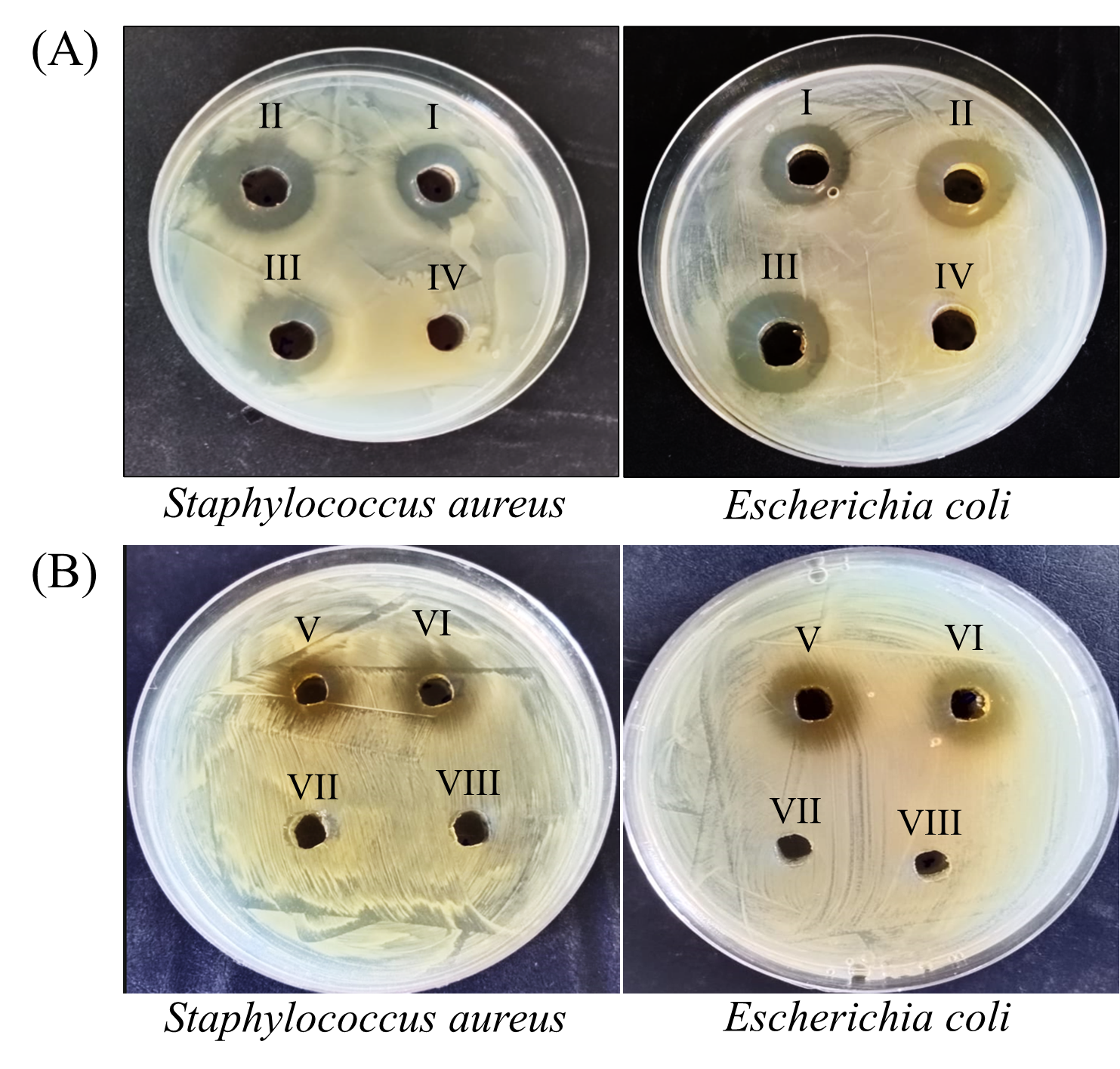


**Figure 4**. *In vitro* anti-microbial activity of sample A and sample B against pathogenic *Escherichia coli* and *Staphylococcus aureus*. (A) Concentrated CFS (65°C, 1.5 hours) from sample A and B produces a clear zone against pathogenic *Escherichia coli* and *Staphylococcus aureus*. Where (I) CFS of sample B; (II) CFS of sample A; (III) CFS of Positive control - *Lactobacillus rhamnosus*; (IV) Negative control – MRS broth. (B) 80% ammonium sulphate precipitation of sample A and B showing inhibitory effect against the pathogenic *E. coli* and *S. aureus*, while no zone was observed in CFS neutralized at pH7. Where (V) partially purified bacteriocin of sample B; (VI) partially purified bacteriocin of sample A; (VII) CFS of sample B at pH7; (VIII) CFS of sample A at pH7.


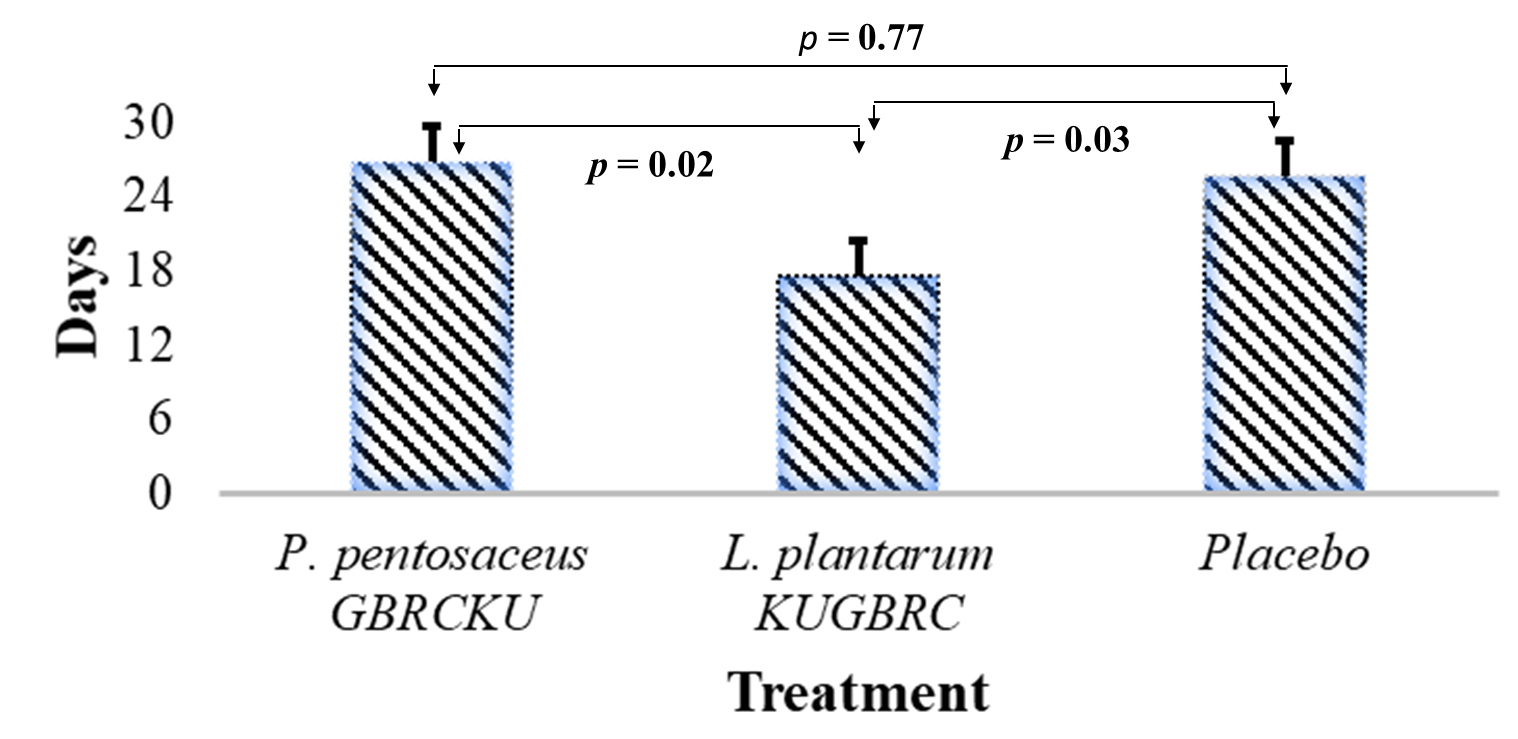


**Figure 5:** *In vivo* Assay: Effect of *P. pentosaceus* GBRCKU (n= 23), *L. plantarum* GBRCKU (n =40) and placebo (n =29) treatment on duration between administration of probiotics to induction of estrus in buffaloes. Line with p value shows the significance level in the graph.

1. **Supplementary Tables**

**Supplementary Table 1. A description of each differentially abundant enzyme between Sample A and Sample B**

| **CAZy families** | **Enzyme family** | **Enzyme name** | **Putative enzymatic activities** | ***L. plantarum* KUGBRC** | ***P. pentosaceus* GBRCKU** |
| --- | --- | --- | --- | --- | --- |
| AA4 | Auxiliary Activity | vanillyl-alcohol oxidase | 1.1.3.38 | 1 | 0 |
| AA10 | Auxiliary Activity | lytic xylan monooxygenase / xylan oxidase (glycosidic bond-cleaving) | 1.14.99.- | 0 | 0 |
| CE20 | Carbohydrate Esterase | xyloglucan acetylesterase (XAC1771) | 3.1.1.6 | 0 | 1 |
| CE2 | Carbohydrate Esterase | acetyl xylan esterase | 3.1.1.72 | 1 | 1 |
| CE4 | Carbohydrate Esterase | peptidoglycan GlcNAc deacetylase | 3.5.1.- | 1 | 0 |
| CE1 | Carbohydrate Esterase | acetyl xylan esterase | 3.1.1.72 | 2 | 0 |
| CE9 | Carbohydrate Estrase | N-acetylglucosamine 6-phosphate deacetylase | 3.5.1.25 | 1 | 2 |
| GH13_29 | Glycoside Hydrolase | α-amylase | - | 1 | 1 |
| GH2 | Glycoside Hydrolase | β-galactosidase | 3.2.1.23 | 1 | 1 |
| GH43_26 | Glycoside Hydrolase | β-xylosidase | 3.2.1.- | 0 | 2 |
| GH170 | Glycoside Hydrolase | 6-phospho-N-acetylmuramidase | 3.2.1.- | 2 | 1 |
| GH125 | Glycoside Hydrolase | exo-α-1,6-mannosidase | 3.2.1.163 | 0 | 0 |
| GH20 | Glycoside Hydrolase | family 20 glycosylhydrolase | 3.2.1.- | 0 | 0 |
| GH85_CB | Glycoside Hydrolase | endo-β-N-acetylglucosaminidase | 3.2.1.96 | 0 | 0 |
| GH42 | Glycoside Hydrolase | β-galactosidase | 3.2.1.23 | 1 | 0 |
| CBM34_GH1 | Glycoside Hydrolase | alpha-glycosidase | - | 1 | 0 |
| GH13_39 | Glycoside Hydrolase | α-amylase | 3.2.1.1 | 1 | 0 |
| GH78 | Glycoside Hydrolase | α-L-rhamnosidase | 3.2.1.40 | 2 | 2 |
| GH38 | Glycoside Hydrolase | α-mannosidase | 3.2.1.24 | 1 | 0 |
| GH25 | Glycoside Hydrolase | lysozyme | 3.2.1.17 | 6 | 2 |
| GH13_31 | Glycoside Hydrolase | α-amylase | - | 4 | 0 |
| GH126 | Glycoside Hydrolase | α-amylase | 3.2.1.- | 0 | 1 |
| GH73 | Glycoside Hydrolase | lysozyme | 3.2.1.17 | 2 | 2 |
| GH13 | Glycoside Hydrolase | maltogenic α-amylase |  | 1 | 0 |
| GH65 | Glycoside Hydrolase | α,α-trehalase | 3.2.1.28 | 4 | 1 |
| GH92 | Glycoside Hydrolase Family | alpha-mannosidase | 3.2.1.- | 0 | 0 |
| GT8 | Glycosyl transferase | peptidase | - | 0 | 2 |
| GT51 | GlycosylTransferase | murein polymerase | 2.4.1.129 | 2 | 2 |
| GT35 | GlycosylTransferase | glycogen or starch phosphorylase | 2.4.1.1 | 1 | 0 |
| GT5 | GlycosylTransferase | UDP-Glc: glycogen glucosyltransferase | 2.4.1.- | 1 | 0 |
| GT26 | GlycosylTransferase | UDP-ManNAcA: β-N-acetyl mannosaminuronyltransferase | 2.4.1.- | 1 | 1 |
| GT28 | GlycosylTransferase | beta-N-acetylglucosaminyltransferase | 2.4.1.- | 1 | 1 |
| GT113 | GlycosylTransferase | Glucosyltransferase | 2.4.1.- | 0 | 1 |

**Supplementary Table 2. Unique genes from pangenome analysis for the strains *P. pentosaceus* GBRCKU and *L. plantarum* KUGBRC**

| **Pfam_ACC** | **Pfam_ID** | **No. of gene count** |
| --- | --- | --- |
| ***P. pentosaceus* GBRCKU** | | |
| PF04371.18 | Porphyromonas-type peptidyl-arginine deiminase | 2 |
| PF03577.18 | Peptidase family C69 | 1 |
| PF01189.20 | 16S rRNA methyltransferase RsmB/F | 1 |
| PF10651.12 | BppU N-terminal domain | 1 |
| PF01380.25 | SIS domain | 2 |
| PF00216.24 | Bacterial DNA-binding protein | 1 |
| PF07852.14 | Protein of unknown function (DUF1642) | 1 |
| PF06114.16 | IrrE N-terminal-like domain | 1 |
| PF07944.15 | Beta-L-arabinofuranosidase, GH127 | 1 |
| PF00440.26 | Bacterial regulatory proteins, tetR family | 1 |
| PF01047.25 | MarR family | 1 |
| PF03613.17 | PTS system mannose/fructose/sorbose family IID component | 1 |
| PF13377.9 | Periplasmic binding protein-like domain | 1 |
| PF03610.19 | PTS system fructose IIA component | 1 |
| PF04326.17 | Putative DNA-binding domain | 1 |
| PF03448.20 | MgtE intracellular N domain | 1 |
| PF13527.10 | Acetyltransferase (GNAT) domain | 1 |
| PF00480.23 | ROK family | 1 |
| PF01370.24 | NAD dependent epimerase/dehydratase family | 1 |
| PF13175.9 | AAA ATPase domain | 1 |
| PF01263.23 | Aldose 1-epimerase | 1 |
| PF05105.15 | Bacteriophage holin family | 1 |
| PF02378.21 | Phosphotransferase system, EIIC | 1 |
| PF12833.10 | Helix-turn-helix domain | 2 |
| PF13333.9 | Integrase core domain | 1 |
| PF00990.24 | Diguanylate cyclase, GGDEF domain | 1 |
| PF01418.20 | Helix-turn-helix domain, rpiR family | 1 |
| PF00185.27 | Aspartate/ornithine carbamoyltransferase, Asp/Orn binding domain | 1 |
| PF13347.9 | MFS/sugar transport protein | 2 |
| PF08282.15 | haloacid dehalogenase-like hydrolase | 1 |
| PF00563.23 | EAL domain | 1 |
| PF00753.30 | Metallo-beta-lactamase superfamily | 1 |
| PF00759.22 | Glycosyl hydrolase family 9 | 1 |
| PF18449.4 | Delta endotoxin | 1 |
| PF13518.9 | Helix-turn-helix domain | 1 |
| PF01261.27 | Xylose isomerase-like TIM barrel | 1 |
| PF13520.9 | Amino acid permease | 1 |
| PF00165.26 | Bacterial regulatory helix-turn-helix proteins, AraC family | 1 |
| PF03830.18 | PTS system sorbose subfamily IIB component | 1 |
| PF06964.15 | Alpha-L-arabinofuranosidase C-terminal domain | 1 |
| PF18423.4 | Zinc binding domain | 1 |
| PF01757.25 | Acyltransferase family | 1 |
| PF20469.1 | Overcoming lysogenization defect protein-like, TOPRIM domain | 1 |
| PF01145.28 | SPFH domain / Band 7 family | 1 |
| PF03609.17 | PTS system sorbose-specific iic component | 1 |
| PF00702.29 | haloacid dehalogenase-like hydrolase | 1 |
| PF14471.9 | Domain of unknown function (DUF4428) | 1 |
| PF00696.31 | Amino acid kinase family | 1 |
| PF01381.25 | Helix-turn-helix | 1 |
| PF13361.9 | UvrD-like helicase C-terminal domain | 1 |
| ***L. plantarum* KUGBRC** | | |
| PF13460.9 | NAD(P)H-binding | 1 |
| PF00005.30 | ABC transporter | 1 |
| PF07724.17 | AAA domain (Cdc48 subfamily) | 1 |
| PF13443.9 | Cro/C1-type HTH DNA-binding domain | 1 |
| PF13354.9 | Beta-lactamase enzyme family | 1 |
| PF10396.12 | GTP-binding protein TrmE N-terminus | 1 |
| PF03235.17 | Protein of unknown function DUF262 | 1 |
| PF02384.19 | N-6 DNA Methylase | 1 |
| PF07693.17 | KAP family P-loop domain | 1 |
| PF01381.25 | Helix-turn-helix | 1 |
| PF13337.9 | Lon-like protease BrxL-like, ATPase domain | 1 |
| PF08747.14 | BREX protein BrxB | 1 |
| PF10097.12 | Predicted membrane protein (DUF2335) | 1 |
| PF08665.15 | PglZ domain | 1 |
| PF02096.23 | 60Kd inner membrane protein | 1 |
| PF08849.14 | BrxA | 1 |
| PF00589.25 | Phage integrase family | 1 |

**Supplementary Table 3. AMR gene found from the *L. plantarum* KUGBRC (Sample A) and *P. pentosaceus* GBRCKU (Sample B)**

|  | **Protein ID, KEGG ID** | |
| --- | --- | --- |
| **Drug class** | **Sample A** | **Sample B** |
| aminoglycoside | M4S29_RS14400 | MCL3858033.1, MCL3858741.1 |
| aminoglycoside | M4S29_RS14401 | MCL3858033.1, MCL3858741.2 |
| aminoglycoside | M4S29_RS14402 | MCL3858033.1, MCL3858741.3 |
| aminoglycoside | M4S29_RS14403 | MCL3858033.1, MCL3858741.4 |
| aminoglycoside | M4S29_RS14404 | MCL3858033.1, MCL3858741.5 |
| aminoglycoside | M4S29_RS14405 | MCL3858033.1, MCL3858741.6 |
| beta-lactam | 0 | 0 |
| beta-lactam | 0 | 0 |
| beta-lactam | 0 | 0 |
| beta-lactam | M00700, K18907, K18104 | 0 |
| beta-lactam | 0 | 0 |
| beta-lactam | 0 | 0 |
| beta-lactam | M00700, K18907, K18104 | 0 |
| beta-lactamase | 0 | 0 |
| cephalosporin | M00700, K18907, K18104 | 0 |
| cephalosporin | M00700, K18907, K18104 | 0 |
| cephalosporin | 0 | 0 |
| cephalosporin | M00700, K18907, K18104 | 0 |
| fluoroquinolone | K18104 | WP_002833736.1, WP_002833389.1 |
| fluoroquinolone | K18104 | WP_002833736.1, WP_002833389.2 |
| fluoroquinolone | K18104 | WP_002833736.1, WP_002833389.3 |
| fluoroquinolone | K18104 | WP_002833736.1, WP_002833389.4 |
| fluoroquinolone | K18104 | WP_002833736.1, WP_002833389.5 |
| glycopeptide | K07260, K08641 | WP_002833736.1, WP_002833389.6 |
| lincomycin | K18104 (imrA) | 0 |
| macrolide | K18231 | 0 |
| nitrofuran | 0 | 0 |
| protein synthesis | 0 | 0 |
| tetracycline | K18220 | 0 |
| Tetracycline | K18220 | 0 |

**Supplementary Table 4. Number of different strains of *Lactiplantibacillus plantarum* and *Pediococcus pentosaceus* retrieved from NCBI to study comparative genome analysis**

| **Name of probiotics** | **Strain name** | **Size (mb)** | **Accession** | **Source** |
| --- | --- | --- | --- | --- |
| *Lactiplantibacillus plantarum* | DF | 36,97,306 | GCA_001953595.1 | *Drosophila melanogaster* |
| *Lactiplantibacillus plantarum* | ATCC 202195 | 33,53,701 | GCA_018351295.1 | Healthy infant faeces |
| *Lactiplantibacillus plantarum* | Heal19 | 33,66,732 | GCA_013367715.1 | vaginal origin to healthy human females |
| *Lactiplantibacillus plantarum* | Y44 | 33,06,722 | GCA_007833595.1 | Fish intense |
| *Lactiplantibacillus plantarum* | CACC 558 | 33,48,896 | GCA_010092485.1 | Canine faeces |
| *Lactiplantibacillus plantarum* | FBL-3a | 32,32,240 | GCA_003999275.1 | Beef cattle faeces |
| *Lactiplantibacillus plantarum* | LQ80 | 34,47,624 | GCA_003097595.1 | Pig feed from feed plant |
| *Lactiplantibacillus plantarum* | LZ206 | 32,63,715 | GCA_001659745.1 | *Bos taurus*  raw cow milk |
| *Lactiplantibacillus plantarum* | DR7 | 32,30,619 | GCA_003586485.1 | *Bos taurus* milk |
| *Lactiplantibacillus plantarum* | plantarum | 31,64,369 | GCA_003076435.1 | weaned piglets |
| *Lactiplantibacillus plantarum* | LZ227 | 34,25,292 | GCA_001660025.1 | *Bos taurus*  raw cow milk |
| *Lactiplantibacillus plantarum* | LB1-2 | 35,41,869 | GCA_002906875.1 | *Apis mellifera* |
| *Lactiplantibacillus plantarum* | AG30 | 34,42,875 | GCA_000687495.1 | Sheep rumen |
| *Lactiplantibacillus plantarum* | Lp1610 | 32,98,660 | GCA_001540925.1 | Teat canal bovine |
| *Lactiplantibacillus plantarum* | MHO2.5 | 3,34,80,971 | GCA_001888495.1 | Shrimp intestine |
| *Lactiplantibacillus plantarum* | A3 | 33,93,963 | GCA_001888525.1 | Marine fish intestine |
| *Lactiplantibacillus plantarum* | JDARSH | 32,01,523 | GCA_003023825.1 | Milk sample |
| *Lactiplantibacillus plantarum* | EML1 | 31,80,629 | GCA_008016845.1 | Wild boar faeces |
| *Lactiplantibacillus plantarum* | SNU.Lp177 | 32,04,772 | GCA_001273585.1 | Porcine faeces |
| *Lactiplantibacillus plantarum* | IMAU80873 | 32,95,567 | GCA_009766165.1 | Acid yak milk |
| *Lactiplantibacillus plantarum* | IMAU20970 | 32,95,567 | GCA_009766165.1 | Acid Camel milk |
| *Lactiplantibacillus plantarum* | NRCC1 | 29,95,133 | GCA_001649985.1 | *Camelus dromedarius* |
| *Lactiplantibacillus plantarum* | ZN-3 | 32,85,934 | GCA_013377705.1 | Chicken gut |
| *Lactiplantibacillus plantarum* | HFC8 | 34,05,709 | GCA_001302645.1 | Human Gut |
| *Pediococcus pentosaceus* | MGBC116430 | 18,94,480 | GCA_910579815.1 | Mouse caecum |
| *Pediococcus pentosaceus* | ET34 | 19,53,235 | GCA_019008315.1 | Salmon |
| *Pediococcus pentosaceus* | SPARC2 | 17,36,516 | GCA_019793535.1 | Animal fecal |
| *Pediococcus pentosaceus* | B4 | 18,35,556 | GCA_011009615.1 | Meles meles fecal sample |
| *Pediococcus pentosaceus* | SMM914 | 19,24,818 | GCA_016652195.1 | Sus |
| *Pediococcus pentosaceus* | TUCO-3 | 19,38,078 | GCA_013385165.1 | Felis catus milk |
| *Pediococcus pentosaceus* | SL001 | 19,19,175 | GCA_007923185.1 | Soil |
